# A who’s who of cell types in skeletal muscle

**DOI:** 10.1038/s42003-026-10803-x

**Published:** 2026-09-03

**Authors:** Shubhangi Das Barman, Zofija Frimand, M. Pilar J. Stella, Anne-Sofie Clausen, Sierra L. W. Olsen, Antoine de Morree

**Affiliations:** Department of Biomedicine, Aarhus University, Aarhus, Denmark

**Keywords:** Cell biology, Molecular biology

## Abstract

Cells are the basic unit of life. In multicellular organisms, cells are organized into tissues. This enables a division of labor where tissues perform complex tasks via coordinated actions of specialized cell types. Accordingly, the cellular collective determines tissue function. A comprehensive overview of which cell types exist in solid tissues is lacking. Using skeletal muscle as a model, we discuss basic principles for cell type classification, summarize 62 unified definitions of cell types in terms of lineage, molecular signatures, and function from the literature, and discuss how these cell types contribute to muscle function. For cell types for which quantitative data was available, we compare abundances in immunohistology and single cell and single-nucleus RNA sequencing data, revealing cell types that are commonly over- or underrepresented in each method. The result is a cell type resource that will serve as a benchmark for single-cell studies of skeletal muscle.

## Abstract (web summary)

In this Review, the authors present distinguishing features for 62 cell types found in skeletal muscle and discuss how each cell type contributes to skeletal muscle function.

## Introduction

Tissue function depends on the coordinated activity of all cell types present in that tissue. To understand how tissue function is regulated, it is key to know which cell types are present in that tissue at a given time, and how they relate to each other. For example, skeletal muscle contraction powers all voluntary movements and enables key body functions, including posture, breathing, speech, and facial expression. But while myofibers give skeletal muscle its contractile ability, myofiber contraction is regulated by interactions with neurons, capillaries, and tendons. Skeletal muscle also serves as a metabolic sink for glucose and amino acids, maintains body temperature, and fosters adaptive immune responses to vaccines. Understanding the regulation of muscle function requires an understanding of the roles of all of its cell types.

Recent advances in tissue dissociation, droplet-based sequencing, and multi-parametric imaging have sprung a large number of single-cell studies that explore the different cell types in a given tissue. These studies resulted in large atlases of molecular cell type definitions. However, while immensely insightful, the level of accuracy and completeness of these cell atlases remains unclear since there is no benchmark to compare them to. Cell type detection and identification can be challenging due to the biological heterogeneity among cell types. For example, large cells may not survive the stringent tissue dissociation procedures that are required to release cells from the tissue; small cells may not be liberated in sufficient numbers to enable confident detection; and adherent cells may not survive in suspension^1–3^. Single nucleus extraction avoids these challenges by omitting the tissue dissociation and cell sorting procedures. Nevertheless, not all nuclei may be equally capable of surviving extraction^4^. Moreover, nuclei mainly yield pre-mRNAs, giving a skewed representation of molecular cell signatures. Even when cells are detected, it can be challenging to assign cell type identities due to the wide range of gene expression levels among cell types. For example, quiescent stem cells have low RNA and protein content, while secretory cells can express orders of magnitude more of specific genes^5,6^. As a result, certain cell type-specific markers may be expressed below the detection limit, while others are expressed at such high levels that their mRNAs diffuse into nearby cells after tissue dissociation - a phenomenon called ambient RNA^7^. Therefore, both low- and high-expressed genes can cause cell misidentification. Cell misidentification also occurs with low-abundant cell types that are too rare to form distinct clusters after dimensionality reduction. Hence, it remains unclear whether single-cell data capture all of the cell types in a tissue at the anticipated relative abundances.

The gold standard for cell type identification is immunohistology. When a cell is observed in the correct anatomical location by staining with an antibody for a cell type-specific protein, one can be certain of its identity. However, this method also comes with limitations. It depends on the availability of specific antibodies and reproducible staining protocols. Moreover, immunohistology works best when a single marker suffices to identify a given cell type, because negative selection is challenging with standard microscopy techniques. Finally, proteins exist in a complex milieu and may require specific processing for detection (i.e., antigen retrieval). As such, spatial information on cell types is often limited. What is needed is a common benchmark of cell types in solid tissues.

**Fig. 1:**
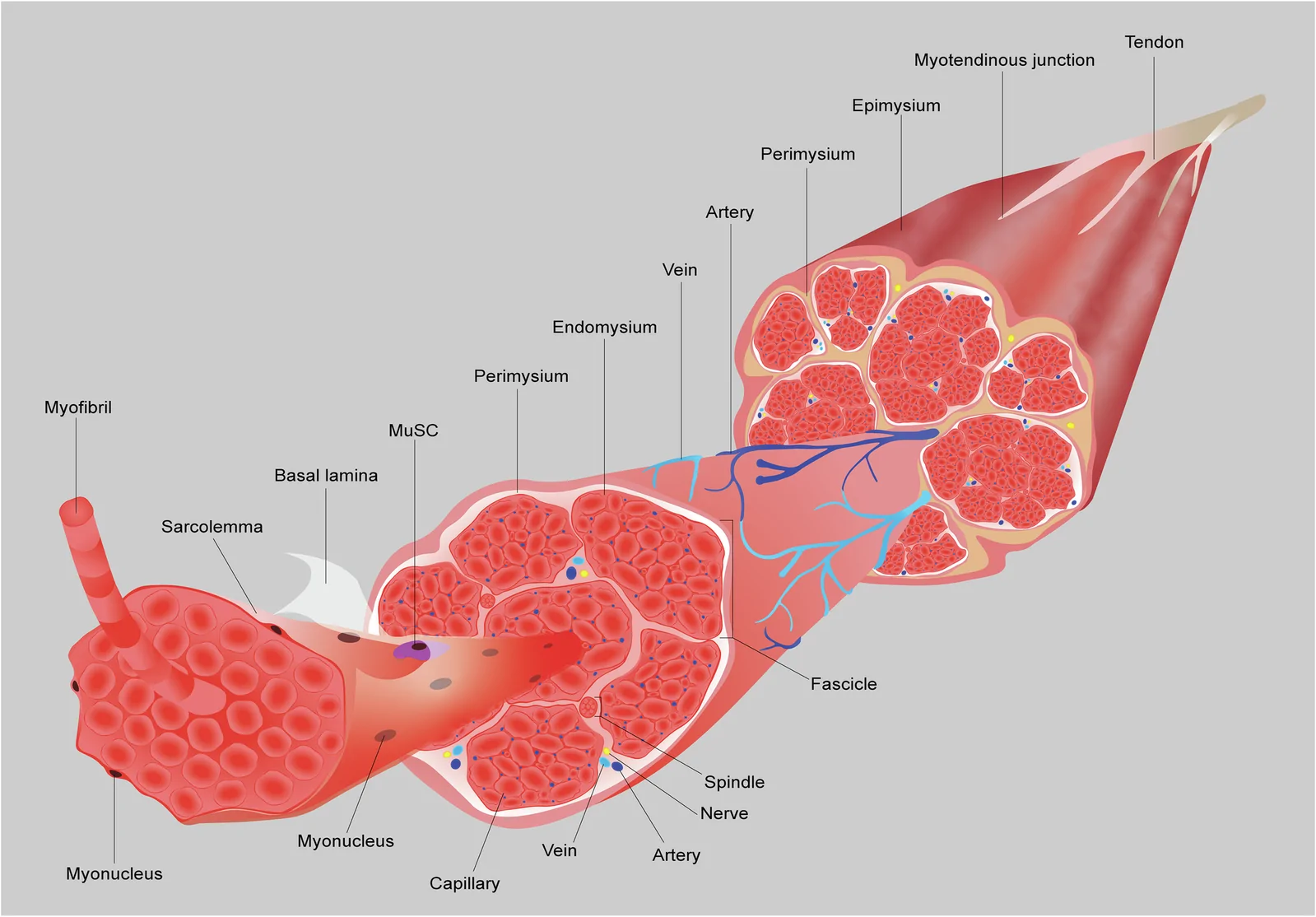
Schematic of skeletal muscle anatomy. Schematic illustrating the spatial organization of skeletal muscle cell types, including the three connective tissue sheets (epimysium, perimysium, and endomysium). Myofibers are bundled in fascicles and bundles of fascicles. Muscle stem cells (MuSCs) localize beneath the basal lamina in direct contact with myofibers. Nerves (light yellow) enter the muscle through the epimysium and terminate in the endomysium. Arteries deliver oxygenated blood (dark blue) via capillaries in the perimysium and endomysium, and veins collect de-oxygenated blood (light blue).

The challenges of cell type detection and identification are apparent in skeletal muscle, a solid tissue that harbors some of the largest and smallest cell types in the body. Skeletal muscles consist of hundreds of myofibers organized by distinct connective tissue sheaths (Fig. 1). Each myofiber is a large multinucleated syncytium wrapped by endomysium, the inner part of which is the basal lamina. The basal lamina also covers small muscle stem cells (MuSCs). Myofibers are bundled together into fascicles and bundles of fascicles wrapped by perimysium (Fig. 1)^8^. In the interstitium between myofibers are a variety of cell types, including vascular and neuronal cells. The muscle is surrounded by epimysium^9^. Here, we provide a comprehensive overview of all cell types that have been reported in human and mouse skeletal muscle and discuss their impact on muscle function. We were especially interested in documenting rare and poorly described cell types, and discussing cell types that plausibly exist in skeletal muscle but are yet to be confidently identified. Finally, we compare cell type abundance based on published RNA sequencing data (single cell (scRNAseq) and single nucleus (snRNAseq)) and immunohistology, revealing cell types that are likely to be over- or under-represented in each method. This comprehensive resource will serve as a benchmark for cell type annotation and single-cell studies of skeletal muscle, and a blueprint for cell type identification in solid tissues in general.

### Cell type definitions

**Fig. 2:**
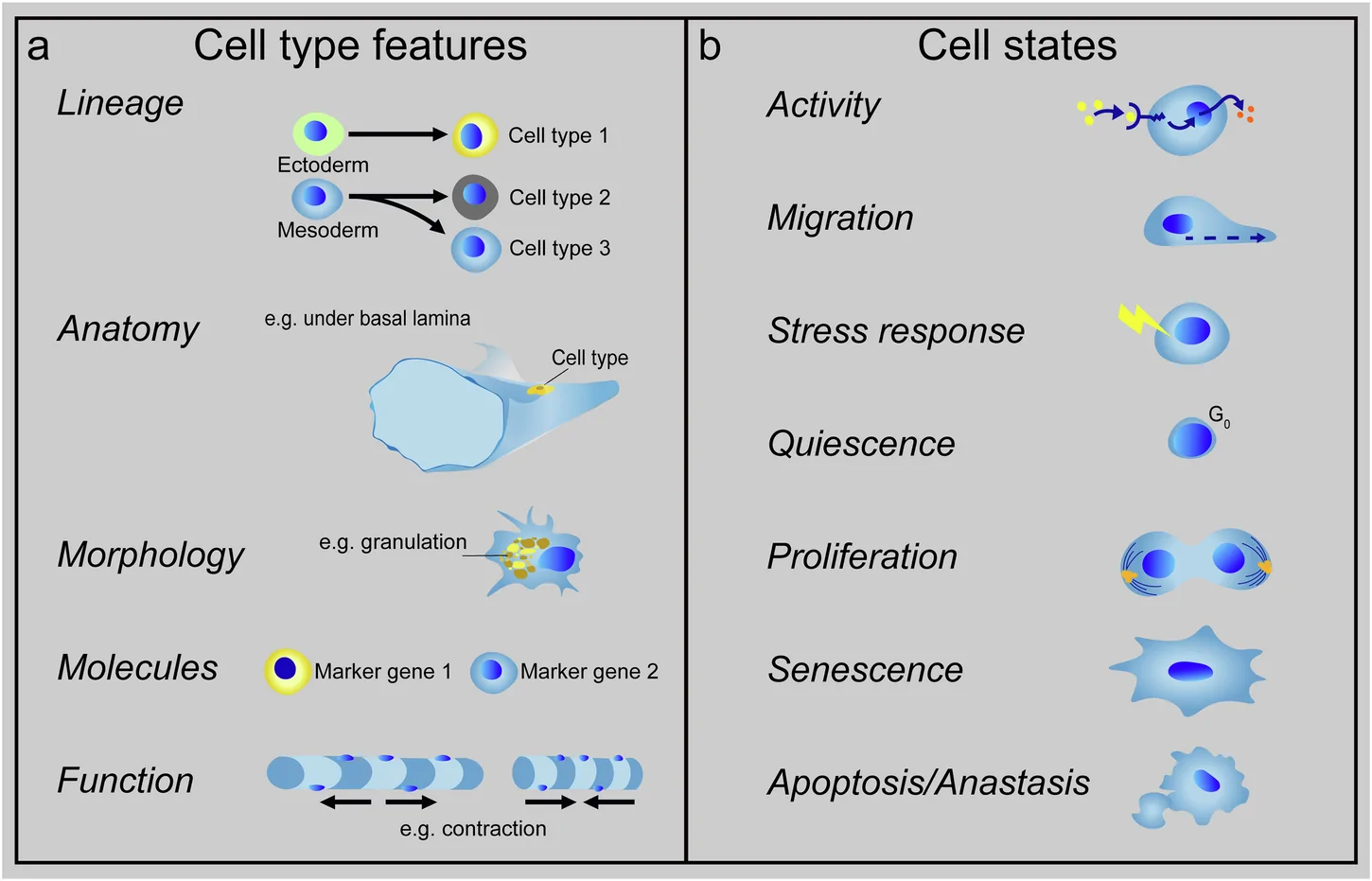
Cell types and states. **a** Cells possess identifiable features that reflect their identity, or type. These features are hardwired and stable. **b** Cells can engage in various processes that reflect their current activity, or state. Cell states are mostly transient and reversible, though they can be irreversible.

**Fig. 3:**
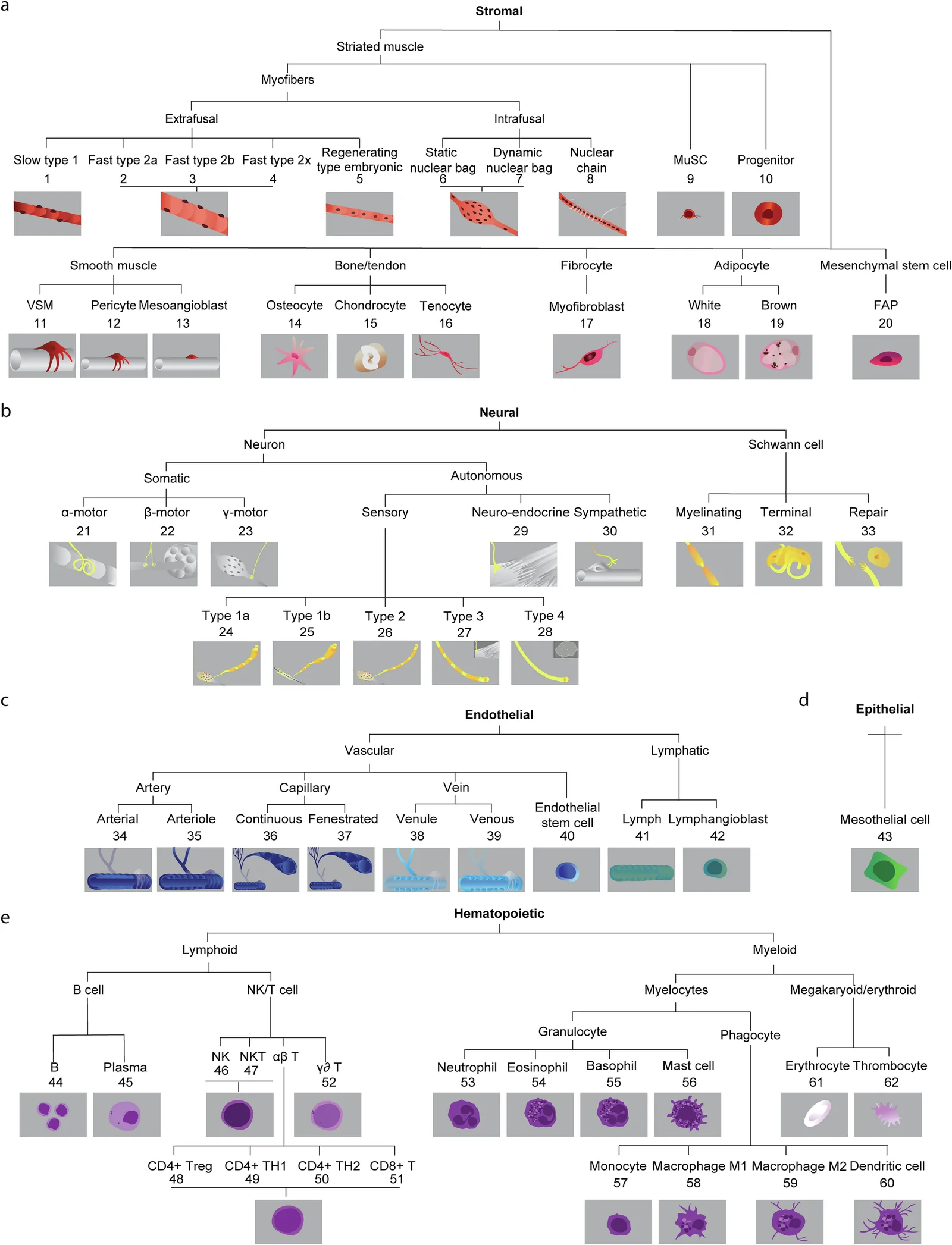
Cell type dendrogram. **a**–**e** Dendrograms of 62 cell types in skeletal muscle. Cell types are grouped by lineage: stromal (**a**), neuronal (**b**), endothelial (**c**), epithelial (**d**), and immune (**e**). Each cell type is numbered. Pictograms illustrate morphological features and anatomical relationships. In those cases where cell types are morphologically similar a single pictogram was used for those cell types.

To find all cell types in skeletal muscle, we first asked what defines a cell type. Although cell categorization is essential for reproducible research, no universal definition of cell types exists^10^. Cells have specific developmental histories, possess identifiable features, and execute specialized functions that can be measured and used to group them into distinct categories of cell identities, called cell types (Fig. 2a)^10^. However, it is often unclear if cell types defined by different features agree with each other, in part because any feature will be heterogeneous across individual cells^10^. Therefore, cell type definitions ideally include multiple features. Cell types can be plastic when cells retain the ability for (de-)differentiation, and they can exist in various cell states. Cell states reflect the various processes a cell may engage in as it responds to its environment (Fig. 2b). The difference between cell type and cell state is not always clear^10^. While cell states do not change cell identities, the extent to which cells engage with processes could contribute to them appearing in a grey zone between cell types, because cells engaging in a particular process will have similarities reflecting that process among them (e.g., expression of cell cycle genes in proliferating cells). We collected descriptions of lineage, function, and features of cells in skeletal muscle and compiled a list of 62 unified cell type definitions (Table 1). We grouped these cell types according to five major lineages to highlight developmental relationships, and graphically represented their morphological features (Fig. 3). Paraxial mesoderm gives rise to 20 stromal cell types (Fig. 3a). Somites from the paraxial mesoderm form the dermomyotome, which gives rise to myogenic cells, and the sclerotome, which gives rise to smooth muscle, connective tissue, and bone cells (Fig. 3a). Ectoderm-derived neural crest cells give rise to 13 neuronal cell types (Fig. 3b). Lateral plate mesoderm gives rise to 9 endothelial cell types that share PECAM1 (CD31) expression and 1 epithelial cell type (Fig. 3c, d). Primitive mesoderm gives rise to immune cells that share PTPRC (CD45) expression (Fig. 3e). The majority of cell types (81%) have been reported in human and mouse muscle, suggesting high conservation of cell types between these species (Supplementary Data). However, cell type marker genes vary between species (Table 1). The remaining cell types are understudied and have been reported in one species only, under specific conditions (11%)(e.g., adipocytes in aged humans), or as a molecular signature that matches a cell type known from other tissues (8%) (e.g., endothelial stem cells).

**Table 1:** Cell type definitions

#	Name	Ontology	Lineage	Anatomy/Morphology	Function	Marker genes [protein name] [unique in Human or Mouse]	Marker protein	Antibody [target]	Ref
1	Extrafusal myofiber, slow, type 1	0002211	Stromal	Long syncytium, peripheral nuclei	Slow contraction	MYH7, TNNC1, TNNI1, TNNT1, TPM3, ACTA1, DES, TTN, DMD, ACTN2, MYOT	MYH7	BA-F8 [MYH7]	^18–21^
2	Extrafusal myofiber, fast, type 2 A	0002214	Stromal	Long syncytium, peripheral nuclei	Fast contraction, metabolism,	MYH2, TNNC2, TNNI2, TNNT3, TPM1, ACTA1, DES, TTN, DMD, ACTN2, MYOT	MYH2	2F7 [MYH2]	^18,21^
3	Extrafusal myofiber, fast, type 2B	0002215	Stromal	Long syncytium, peripheral nuclei	Fast contraction, metabolism,	MYH4, TNNC2, TNNI2, TNNT3, TPM1, ACTA1, DES, TTN, DMD, ACTN2, MYOT	MYH4	10F5 [MYH4]	^18,21^
4	Extrafusal myofiber, fast, type 2X	4052026	Stromal	Long syncytium, peripheral nuclei	Fast contraction, metabolism,	MYH1, TNNC2, TNNI2, TNNT3, TPM1, ACTA1, DES, TTN, DMD, ACTN2, MYOT	MYH1	6H1 [MYH1]	^18,21^
5	Extrafusal myofiber, type regenerating	0009097	Stromal	Long syncytium, central nuclei	Muscle repair	MYH3, ACTA1, DES, TTN, DMD, ACTN2, MYOT	MYH3	F1.652 [MYH3]	^18^
6	Intrafusal myofiber, static nuclear bag	4023006	Stromal	Short, bulge of nuclei	Proprioception	MYL2, MYH7, ACTA1, DES, TTN, DMD, ACTN2, MYOT	MYL2		^25,26^
7	Intrafusal myofiber, dynamic nuclear bag	4023005	Stromal	Short, bulge of nuclei	Proprioception	MYL2, MYH7. MYH8, ACTA1, DES, TTN, DMD, ACTN2, MYOT	MYL2		^25,26^
8	Intrafusal myofiber, nuclear chain	4023003	Stromal	Short, chain of nuclei	Proprioception	MYL2, MYH3, MYH6, MYH8, ACTA1, DES, TTN, DMD, ACTN2, MYOT	MYL2, MYH6	S46 [MYH6]	^25,26^
9	Skeletal muscle satellite stem cell	0008011	Stromal	Sub-basal lamina, myofiber-adjacent	Muscle repair & maintenance	PAX7, MYF5, CALCR, MYOD1[M], NCAM1[CD56], CD82[H], VCAM1[M], ITGA7, CXCR4	PAX7	Pax7c [PAX7]	^5,34–36^
10	Myogenic progenitor cell	0000680	Stromal			MYOD1, MYOG, MYF6, CD9, ITGB4 [CD104]	CD9	MZ3 [CD9]	^45,46^
11	Vascular smooth muscle myoblast	0000514	Stromal	Perivascular	Vascular resistance	TAGLN, ACTA2[SMA], ACTG2, DES, SMTN, CNN1, MYH11, MYOCD, PDGFRB, RGS5, HEYL, NOTCH3, TINAGL1	SMA	D4K9N [SMA]	^139^
12	Pericyte	0000669	Stromal	Perivascular	Vascular resistance	TAGLN, ACTA2[SMA], DES, SMTN, PDGFRB, CSPG4 [NG2], RGS5, ABCC9, KCNJ8, CD248, DLK1, TRPC6, HIGD1B, NOTCH3	NG2		^138,141^
13	Mesoangioblast	0000566	Stromal	Perivascular		MCAM[CD146], PDGFRA[CD140A], PDGFRB[CD140B], CD44, CSPG4[NG2], ACTA2[SMA], ANXA5, DES	CD146, NG2	D4K9N [SMA]	^143,144^
14	Osteocyte	0000137	Stromal			RUNX2[CBFA1], SP7[OSX], ALPL, SPP1, BGLAP[OCN], ALPL[AP], SPARC, IBSP	OSX		^77–79^
15	Chondrocyte	0000138	Stromal			COL2A1, COMP, OGN, MGP, ACAN, SOX9, SCIN, HAPLN1	SOX9		^77–79^
16	Tenocyte	0000388	Stromal	Interstitium, near tendon	Tendon support	SCX, THBS4, COMP, TNC, MKX, COL1A1, COL1A2, COL3A1, FMOD, TNMD [M]	SCX		^48–51^
17	Myofibroblast	0000186	Stromal			PDGFRA, COL1A1, LUM, TAGLN, ACTA2, ACTG2, WIF1, FGF18, POSTN	ACTA2 [SMA]		^76,157^
18	White adipocyte	0000448	Stromal	Interstitium, near tendon		ADIPOQ, LPL, PLIN1, FABP4, PNPLA2, CEBPA, PPARG, AOC3, IGF1, ACSL5, FASN, APOE	PLIN1	D1D8 [PLIN1	^65,69^
19	Brown adipocyte	0000449	Stromal			CIDEA, CIDEC, P2RX5, UCP1, PRDM16, LETMD1, ZIC1	UCP1		^71,74,75^
20	Fibroadipogenic progenitor cell	0009099	Stromal	Interstitium	Muscle repair & maintenance	PDGFRA, CD34[M], LY6A[SCA1][M], THY1[CD90], TCF7L2[H]	PDGFRA		^37,56,57,59^
21	Motor neuron, alpha	0008038	Neuronal		Voluntary contraction	RBFOX3, FBXL7, KCND3, ZEB2, GRM5, KCNT2, KITLG [SCF], DPYD, FBN2, FGF13, VIPR2, SPP1	RBFOX3		^85–87^
22	Motor neuron, beta	4023000	Neuronal		Voluntary contraction	GPR149, RREB1	GPR149		^84^
23	Motor neuron, gamma	0008037	Neuronal		Voluntary contraction	ESRRG, ESRRB, GFRA1, KCNA5, LPP, NFIA, NR3C2, RET, TNS1, SH3PXD2A, PLEKHG1	ESRRB		^89–92^
24	Sensory neuron, type 1a	1001451	Neuronal	Thick myelinated	Proprioception	WHRN, PVALB, RUNX3, NTRK3, ETV1, LMCD1, NTRK3[TRKC]	PVALB		^25,96,97^
25	Sensory neuron, type 1b	1001451	Neuronal	Thin myelinated	Proprioception	WHRN, PVALB, RUNX3, NTRK3, ETV1, CHAD, POU4F3[BRN3C]	PVALB		^25,96,99,100^
26	Sensory neuron, type 2	1001451	Neuronal	Thick myelinated	Proprioception	WHRN, PVALB, RUNX3, NTRK3, ETV1, FXYD7	PVALB		^25,96,99^
27	Sensory neuron, type 3	0000102	Neuronal	Thin myelinated	Noci-, thermoception	CGRP, TAC1 [substance P]	CGRP		^25,102^
28	Sensory neuron, type 4	0000102	Neuronal	Non-myelinated	Noci-, thermoception	CGRP, TAC1 [substance P]	CGRP		^25,102^
29	Neuro-endocrine cell	0000165	Neuronal	Nerve end at NMJ		CHGA, CHGB, TAC1, TPH1, CPE, NEUROD1	CHGA		^93,94^
30	Sympathetic neuron	0011103	Neuronal	Non-myelinated	Blood flow	TH	TH		^108^
31	Myelinating Schwann cell	0002573	Neuronal	Along peripheral nerves	Myelination	ERBB3, S100B, PLP1, SOX10, MBP, MPZ, PTN, CRYAB, NCMAP, PLLP, PRX, MAG, EGR2	MBP		^109^
32	Terminal Schwann cell	0000692	Neuronal	At NMJ	NMJ protection	ERBB3, S100B, PLP1, SOX10, NGFR, SCN7A, APOD, SMOC2, ECM1, NCAM1, L1CAM, NRXN1			^111^
33	Repair Schwann cell	0002375	Neuronal			JUN, NCAM1, GFAP			^111^
34	Artery endothelial cell	1000413	Endothelial	Artery lining	Vascular resistance	GJA5, BMX, GJA4, EFNB2, DKK2, CXCL12, SOX17, HEY1, FBLN5, KDR[VEGFR2], PECAM1			^122–124,129^
35	Arteriole endothelial cell	1000412	Endothelial	Arteriole lining	Vascular resistance	PSP4, DES, DSTN, CNN1, TGFB2, GLUL, PECAM1			^122–124^
36	Capillary endothelial cell, continuous	0002144	Endothelial	Capillary lining	Exchange of molecules & cells	CA4[CAHA4], AQP7, GPIHBP1, RGCC, PRX, VWA1, EDN1, EPAS1, ADGRE5, NTRK2, MFSD2A, PECAM1	CA4		^116,117^
37	Capillary endothelial cell, fenestrated	0000666	Endothelial	Capillary lining with fenestrae	Exchange of molecules & cells	CA4[CAHA4], AQP7, GPIHBP1, RGCC, PRX, VWA1, EDN1, EPAS1, ADGRE5, NTRK2, MFSD2A, PLVAP[PV1], PECAM1	PLVAP		^116–118^
38	Venule endothelial cell	1000414	Endothelial	Venule lining	Vascular resistance	TFRC, CAR4, MADCAM1, LRG1, ACKR1, PECAM1			^206^
39	Vein endothelial cell	0002543	Endothelial	Vein lining	Vascular resistance	ACKR1[DARC], NR2F2, VCAM1, SELP, ACKR3, IL1R1, PRCP, PTGS1, RGS5, EPHB4, SLC38A5, PECAM1			^125–128^
40	Endothelial stem cell	0002619	Endothelial			KIT, PECAM1	KIT		^135^
41	Lymph endothelial cell	0002138	Endothelial	Lymphatic lining	Fluid drainage	PROX1, LYVE1, FLT4, PDPN, CCL21, SEMA3A, TBX1, NR2F1, KDR [VEGFR2], TEK, RELN	LYVE1		^130–133^
42	Lymphangioblast	0005020	Endothelial			CD34, LYVE1^low^, PROX1^low^	CD34		^136^
43	Mesothelial cell	0000077	Epithelial	Mesothelial lining		MSLN, UPK3B, PTGIS, IGFBP6, NKAIN4 [M], LRRN4, WT1, CALB2, BNC1	MSLN		^194,195^
44	B cell	0000236	Immune		Immune memory	CD19, MS4A1[CD20], CD22, FCER2[CD23], CD24, CD72, CD79A, CD79B	CD19, CD20	SP32 [CD20]	^166^
45	Plasma cell	0000786	Immune		Antibody production	SDC1[CD138], JCHAIN, TNFRSF17[BCMA], PRDM1[BLIMP1], XBP1, CD38[H], CD28[M]	CD138		^157,168^
46	Natural killer cell	0000623	Immune			CD2, KLRB1[NK1.1], KLRD1[CD94], KLRF1 [NKP80], NCR1 [NKP46], FCER1G, NKG7, GZMA, GNLY[H], CD27[M], NCAM1[CD56][H], FCGR3A[CD16][H], ITGAM[CD11B][M]			^157,158^
47	Natural killer T cell	0000814	Immune			CD2, CD3D, CD3E, KLRB1[CD161][NK1.1], KLRD1[CD94]			^157,158^
48	T cell, regulatory	0000815	Immune		Immune suppression	CD2, CD3D, CD3E, CD4, FOXP3, IL2RA [CD25], CTLA4, TNFRSF18 [GITR]	FOXP3		^150,151^
49	T cell, CD4, helper 1	0000545	Immune		Immune memory	CD2, CD3D, CD3E, CD4, CXCR3 [H], TBX21, IFNG			^149^
50	T cell, CD4, helper 2	0000546	Immune		Immune memory	CD2, CD3D, CD3E, CD4, IL4, IL5, IL13, CRTH2 [H], GATA3			^149^
51	T cell, CD8	0000795	Immune		Cell clearance	CD2, CD3D, CD3E, CD8A	CD8	53-6.7 [CD8A]	^154^
52	γδ T cell	0000798	Immune			CD2, CD3D, CD3E, CD3G, TRDC, TRGC1, TRGC2,			^163,164^
53	Neutrophil	0000775	Immune		Cell clearance	ITGAM[CD11B], SELL[CD62], S100A8, S100A9, S100A12, CSF3R, GFI1, ELANE, MPO, AZU1, ARG1, MME, CAMP, LTF, MMP9, ALPL, LY6G[M], FUT4[CD15][H], FCGR3A[CD16][H]			^155,177–180^
54	Eosinophil	0000771	Immune			IL5RA, CCR3, CCR1, ADGRE1 [EMR1], SIGLEC8, GATA1, EPX, PRG2, RNASE2, SIGLECF[M]			^182,183^
55	Basophil	0000767	Immune			FCER1A, HDC, MS4A2, GATA2			^157,186^
56	Mast cell	0000097	Immune	Peri-, endomysium		TPSB2, HDC, TPSAB1, CPA3, MCPT4 [M], MCPT5 [M], MCPT6 [M], MCPT8 [M], FCER1A			^187,188^
57	Monocyte	0000576	Immune			CSF1R, VCAN, S100A8, S100A12, CD14 [H], FCGR3A [CD16] [H], LY6C1 [M], LY6C2 [M]			^157^
58	Macrophage, type 1	0000863	Immune			TNFRSF1B[CD120B], TNF,TIMD4[TIM4], FCGR3A[CD16][H], LY6C1[M], IL1B, CD68, CD80, IL6			^173,174^
59	Macrophage, type 2	0000890	Immune			CD1A, CD93, CD226, MARCO,MRC1, CD163, C1QA, CD68, ADGRE1[EMR1], ITGAM[CD11B][M]			^173,174^
60	Dendritic cell	0000451	Immune		Antigen presentation	FLT3, CCL19, CCR7, CCL17, CCL22, FSCN1, LAMP3, IRF8, ITGAX [CD11C]			^155,169^
61	Erythrocyte	0000232	Immune	Blood vessel lumen	Oxygen delivery	GYPA [CD235A], TFRC [CD71], ALAS2, AHSP, GATA1, HBA1, HBB			^190^
62	Thrombocyte	0000233	Immune	Blood vessel lumen	Hemostasis	ITGA2B [CD41], GP9, VWF, PF4, PPBP, GATA1, TUBB1, MYL9, SELP, GP6, GP1BA, GP1BB			^189^

Overview of cell types in skeletal muscle, following the numbering in Fig. 2. For each cell type, the table lists the name, Cell Ontology number, lineage, function, the features (anatomical/morphological, RNA, protein), and validated antibody clones.

### Cell microenvironments

**Fig. 4:**
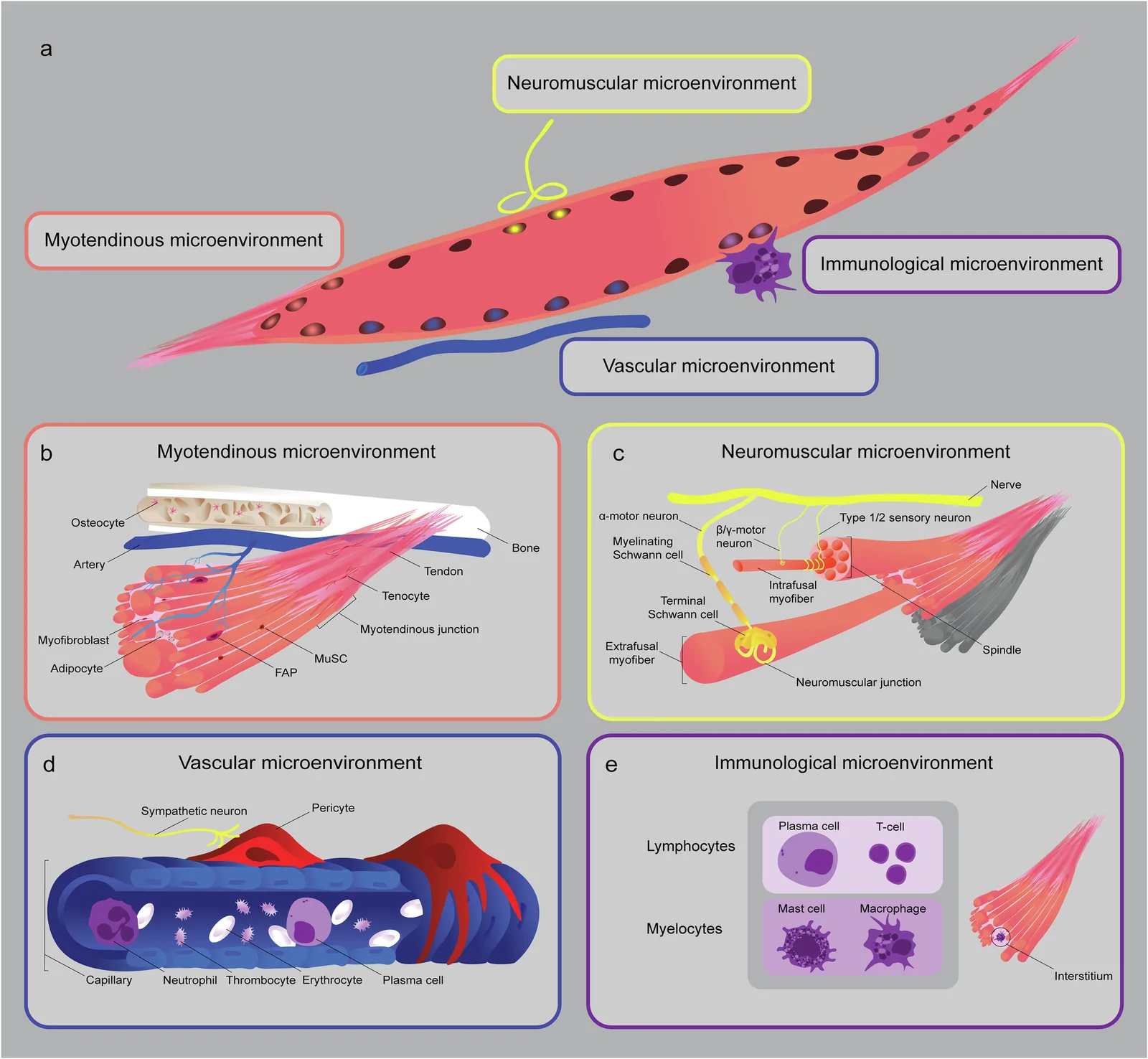
Schematic of anatomical structures in skeletal muscle. **a** Schematic overview of a myofiber and its peripheral myonuclei, with four key anatomical microenvironments highlighted. **b**–**e** Schematic representations of the four microenvironments from (**a)**. Shown are the myotendinous (**b**), neuromuscular (**c**), vascular (**d**), and immunological (**e**) microenvironments, with key cell types highlighted in each.

The 62 cell types together regulate skeletal muscle function by interacting with each other in compartmentalized microenvironments. These microenvironments are defined anatomically and leave distinct molecular signatures in subsets of myonuclei (Fig. 4a). Myonuclei near myotendinous junctions express genes to regulate the myotendinous junction in response to interactions between the myofiber and the surrounding matrix and nearby myotendinous cells (Fig. 4a, b)^11,12^. A second microenvironment occurs at sites of innervation. Myonuclei near neuromuscular junctions (NMJs) specifically express genes for post-synaps formation in response to interactions with innervating neurons (Fig. 4a, c)^13^. Myonuclei also respond to interactions with the vascular network (Fig. 4a, d). Such interactions are mediated by secreted molecules and affect fiber type and angiogenesis^14^. Finally, secreted molecules mediate interactions between myofibers and immune cells, resulting in distinct local transcriptional signatures (Fig. 4a, e)^12,15^. In the next section, we discuss the 62 cell types in light of their roles in skeletal muscle function and we organize cell types by these microenvironments (Fig. 4b–e).

### Cell type function

#### Musculature

The quintessential cell type in skeletal muscle tissue is the extrafusal myofiber (Figs. 1 and 3a). Myofibers are multinucleated syncytia that can be up to 100 µm in diameter and up to dozens of cm long. On the far ends, they connect to tendons via myotendinous junctions. Where human myofibers typically run the length of the muscle, mouse myofibers often end intrafascicularly^16,17^. Myofibers are characterized by peripheral myonuclei and central myofibrils, which make up the contractile machinery. They are molecularly defined by expression of skeletal muscle actin (ACTA1) and genes encoding the contractile apparatus (MYOT, TTN)(Table 1)^18,19^. Extrafusal myofibers can be subclassified based on gene expression and contractile properties (Fig. 3a). Type I (slow-twitch) myofibers express myosin heavy chain 7 (MYH7), troponins C1, I1, and T1 (TNNC1, TNNI1, TNNT1), tropomyosin 3 (TPM3), and oxidative phosphorylation genes (PPARGC1A) (Table 1)^18,20,21^. The proteins encoded by these genes enable sustained, low-force contractions and endurance activities. Type II (fast-twitch) myofibers express TNNC2, TNNI2, TNNT3, and TPM1^18,21^. They can be further subclassified based on myosin expression in type IIA (MYH2), IIX (MYH1), and IIB (MYH4) myofibers, with increasingly faster contractile properties (Table 1)^18,21^. Type II myofibers enable activity bursts. Importantly, while myofiber classification based on myosins works well for mouse muscles, it is less applicable to human muscles, which contain intermediate myofibers that express multiple myosins^22^. Finally, regenerating myofibers are thin and marked by central myonuclei and MYH3 expression (Table 1)^18^. Extrafusal myofibers can change myosin expression in response to changes in nerve activity. Because such fiber type switches only occur in response to prolonged changes in activity^23^, we consider fiber types as cell types and not cell states.

Skeletal muscle also contains intrafusal myofibers within proprioceptive organs called muscle spindles (Figs. 1 and 3a). Muscle spindles have a distinct connective tissue sheath filled with viscous fluid and are embedded between fascicles (Fig. 4b)^24^. Compared to extrafusal myofibers, intrafusal myofibers are shorter and thinner. They can be morphologically subclassified as nuclear chain, static nuclear bag, and dynamic nuclear bag myofibers, which all express MYL2 and pan-myofiber markers (Table 1)^25^. There are currently no unique markers to distinguish intrafusal myofiber subtypes. However, human nuclear chain fibers express MYH3, MYH6, and MYH8, while static nuclear bag fibers express MYH7 and dynamic nuclear bag fibers express MYH7 and MYH8^26^. Intrafusal fibers play critical roles in proprioception by detecting changes in muscle length and tension^27^. This sensory feedback enables fine motor control and spindle density is highest in muscles involved in fine, precise movement^24,28^.

Due to their remarkable length and repeated contractions, myofibers are highly susceptible to damage. Unsurprisingly, skeletal muscles possess mechanisms to repair such damage. First, myofibers can self-repair membrane tears and sarcomere breaks, and a lack of either mechanism causes muscular dystrophy^29,30^. Second, myofibers can be regenerated from MuSCs. MuSCs were first defined as satellite cells due to their anatomical position, juxtaposed on a myofiber under the basal lamina (Fig. 1)^31^. They associate with extrafusal and intrafusal myofibers and have a heterochromatic nucleus and long protrusions^32,33^. PAX7 is the canonical marker, but MuSCs also express NOTCH target genes and CALCR^5,32^. During homeostasis, MuSCs reside in a quiescent state of prolonged reversible cell cycle exit^5^. The quiescent state is regulated post-transcriptionally and quiescent MuSCs express mRNA for the activation genes MYF5 and MYOD1, but not the protein (Table 1)^5,34–36^. Depletion of PAX7^+^ cells abolishes the muscle’s ability to regenerate^37–39^. In addition, MuSCs may play roles in hypertrophy of extrafusal myofibers and maintenance of extrafusal and intrafusal myofibers in some, but not all, muscles^40–44^. Finally, mouse skeletal muscle harbors myogenic progenitor cells, committed descendants of MuSCs expressing CD9 and CD104 (Table 1)^45,46^. Their function remains poorly understood.

#### Myotendinous microenvironment

When muscles contract, they transmit force through tendons (Fig. 4b). Tendons primarily connect muscle to bone, but they can also connect to other muscles, fascia, skin or the eyeball. Tendons consist of large collagen fibrils of up to 300 µm in diameter that extend into the belly of the muscle, where they flatten out to form the aponeurosis, a variable extension that consists of smaller collagen fibers to which most of the myofibers connect^47^.

The primary cell type in the tendon area is the tenocyte (Figs. 3a and 4b). Tenocytes localize between collagen fibrils within the interfascicular matrix. Mature tenocytes are elongated cells with long, thin protrusions. They express scleraxis (SCX), tenascin C (TNC), and mohawk (MKX)^48,49^. Notably, KRT7 and tenomodulin (TNMD) uniquely mark human and mouse tenocytes, respectively (Table 1)^50,51^. Tenocytes produce extracellular matrix (ECM) components essential for tendon maintenance and repair. Depletion of SCX^+^ cells leads to defective muscle attachment and germline deletion of SCX impairs tendon growth and repair^49,52,53^. However, a recent study suggests that SCX is transiently expressed in activated MuSCs^54^, indicating that the deletion studies should be interpreted with caution.

Fibroadipogenic progenitors (FAPs), a type of mesenchymal stem cell, are present throughout the interstitium outside the capillary basement membrane and basal lamina (Fig. 4b). They express mesenchymal stem cell markers THY1 and NT5E, and stromal markers PDGFRA, TCF4, and HIC1. Unlike human FAPs, mouse FAPs express LY6A and CD34 (Table 1)^37,55–60^. During homeostasis, FAPs reside in a quiescent state^5,56,57^. Upon injury, they proliferate and can differentiate into adipocytes, myofibroblasts, chondrocytes, osteocytes, and possibly tenocytes^56,57,60,61^. FAPs play central roles in muscle homeostasis by supporting MuSCs and modulating immune cell recruitment^62^. Depletion of PDGFRA^+^ cells results in muscle atrophy and defective repair, and depletion of TCF4^+^ cells compromises muscle repair^37,63^. In contrast, HIC1 deletion causes FAP activation and defective muscle repair^60^.

Adipocytes are common in muscles of aged humans—but not aged mice—and in various pathological contexts such as muscular dystrophies and cardiometabolic diseases^64,65^. They reside in the interstitial space near the myotendinous area (Figs. 3a and 4b)^66,67^. Adipocytes can be subclassified. White adipocytes are the predominant adipocytes in aged human muscle^68^. They contain a single lipid droplet and express adiponectin (ADIPOQ), perilipin (PLIN1), and leptin (LEP) (Table 1)^65,69^. Their function in skeletal muscle is unclear, but in other tissues they enable fat storage and adipokine signaling. Brown adipocytes have not been observed in healthy skeletal muscle but appear in mouse muscle upon injury^70^. These cells share a developmental origin with MuSCs and can be derived ex vivo from FAPs^70–73^. Brown adipocytes contain multiple lipid droplets, a high mitochondrial content, and uncoupling protein 1 (UCP1), CIDEA, and PRDM16 (Table 1)^71,74,75^. Their function in skeletal muscle remains unknown, but in brown adipose tissue, they mediate thermogenesis and batokine signaling.

Myofibroblasts are contractile cells near tendons that express collagens, smooth muscle actin (ACTA2), and signaling molecules (IL7) (Fig. 3a)^76^. Although their function in muscle remains undefined, their molecular profile suggests roles in extracellular matrix formation and immune regulation. They accumulate with age. Chondrocytes and osteocytes produce matrix and calcium deposits, respectively. Chondrocytes express commitment factor SOX9 and ECM proteins CNMD and HAPLN1, whereas osteocytes express osterix (SP7) and osteopontin (SPP1) (Table 1)^77–79^. Although neither cell type has been reported in healthy muscle, they appear in response to bone/muscle fractures or inflammation, respectively^80,81^.

#### Neuromuscular microenvironment

Skeletal muscle contraction is voluntary and initiated by nerves via excitation-contraction coupling and regulated by feedback from sensory neurons (Fig. 4c).

Myofibers are innervated by motor neurons (Figs. 3b and 4c). Motor neurons have their soma in the ventral horn of the spinal cord or brainstem, and extend long myelinated axons into the muscle to terminate at NMJs (Fig. 4c)^82^. (We did not find any reports of neuronal somas inside muscle tissue, analogous to the neuronal somas in the gut^83^.) While each myofiber is innervated by a single motor neuron, each motor neuron innervates multiple myofibers. The group of myofibers innervated by a single motor neuron is called a motor unit^82^. Motor neurons express choline acetyltransferase (CHAT) and SV2B, and release acetylcholine into the NMJ to trigger myofiber contraction^84^. Motor neurons can be subclassified based on anatomy, marker expression, and function. ɑ-Motor neurons innervate extrafusal myofibers and express RBFOX3, VIPR2, and SPP1 (Figs. 3b and 4c, and Table 1)^85–87^. Loss of ɑ-motor neurons leads to muscle atrophy^88^. β-motor neurons innervate both intrafusal and extrafusal myofibers and express markers such as GPR149 and RREB1 (Figs. 3b and 4c, and Table 1)^84^. They ensure that the intrafusal myofibers contract in sync with the extrafusal myofibers. ɣ-Motor neurons innervate intrafusal myofibers and express ESRRG, ESRRB, and the GDNF receptor GFRA1 (Figs. 3b and 4c, and Table 1)^89–92^. ɣ-Motor neurons are selectively lost after germline deletion of GDNF, but this does not affect intrafusal fiber size or ambulation, possibly because the β-motor neurons can compensate^90^.

In contrast to motor neurons, neuroendocrine cells secrete neurotransmitters and neurohormones beyond the NMJ (Fig. 3b). They have been documented in the rat diaphragm near cholinergic nerve endings and express chromogranin A (CHGA), a pro-protein that can be processed into several bioactive peptides^93,94^. CHGA knockout mice show reduced muscle mass and exercise-induced regeneration^94^. To what extent this depends on the loss of neuroendocrine function in skeletal muscle remains unknown.

Upon muscle contraction, sensory neurons relay information back to the central nervous system (Figs. 3b and 4c). Sensory neurons have their somas in the dorsal root ganglion, and their dendrites terminate in the muscle. They express PIEZO2 and ATP1A3^25^. A lack of sensory neurons results in ataxic gait and absence of tendon reflexes^95^. Sensory neurons can be subclassified anatomically and functionally. Type 1 and Type 2 sensory neurons innervate intrafusal myofibers with annulospiral endings (Figs. 3b and 4c, and Table 1)^28^. Type 1a sensory neurons are thick, densely myelinated, and marked by LMCD1 and TRKC expression^96,97^. They sense changes in muscle length and contraction speed^98^. Germline loss of TRKC results in a loss of Type 1a sensory neurons and an inability of mice to maintain an upright posture on a rotating dowel^97^. Type 1b sensory neurons specifically innervate muscle spindles in the tendon area, also called Golgi tendon organs, and express POU4F3 and CHAD^96,99,100^. They sense muscle tension. Germline loss of ER81 leads to a loss of Type 1b sensory neurons and the absence of Golgi tendon organs, causing the mice to move slower^101^. Type 2 sensory neurons are thin, densely myelinated, and primarily innervate nuclear chain fibers. They express FXYD7 and exhibit oscillatory firing patterns in the absence of movement^96,99^. Type 3 and Type 4 sensory neurons have free sensory nerve endings that do not terminate at specialized receptive structures (Fig. 3b)^102^. These cells enable nociception and thermoception^102,103^. Type 3 sensory neurons have a thin myelin sheath, whereas Type 4 dendrites are non-myelinated^102^. Both cell types are marked by CGRP and substance P^102^, but molecular separation is not yet possible. Type 3 and 4 sensory neurons respond to metabolites (i.e., lactate), low pH or thermal stimuli and thus play key roles in sensing muscle fatigue and pain^102–104^.

Skeletal muscle is also influenced by the visceral nervous system^105^. The non-myelinated axons of sympathetic neurons are present along arteries and reach into the muscle to terminate near perivascular cells (Fig. 4d)^106,107^. Sympathetic neurons are marked by tyrosine hydroxylase (TH) (Table 1)^108^. Administration of the toxic dopamine analog 6-OHDA results in sympathetic denervation, reduced pericyte coverage of muscle capillaries, and reduced blood flow^107^. Moreover, noradrenaline secreted by sympathetic neurons triggers angiopoietin secretion from pericytes, which signals MuSCs to remain quiescent. Sympathetic denervation results in MuSC activation and fusion^107^.

Most peripheral neurons are protected by Schwann cells, a glial cell in the peripheral nervous system (Figs. 3b and 4c). Schwann cells wrap around axons and proprioceptive dendrites in a spiral fashion to form a protective myelin sheath that enables rapid transmission of electrical signals^109^. Schwann cells express S100, SOX10, and myelin basic protein (MBP) (Table 1)^109^. Schwann cells at the NMJ do not make myelin or wrap around axons but instead orient themselves longitudinally to cover the nerve terminals and are called terminal Schwann cells^110^. They express S100, but not MBP, and maintain NMJ integrity (Table 1)^111^. In response to nerve injuries, myelinating and terminal Schwann cells can dedifferentiate into repair Schwann cells. Repair Schwann cells express c-JUN and promote axon survival, axon growth, and re-establishment of neuromuscular connections^112,113^.

#### Vascular microenvironment

Skeletal muscles rely on an extensive capillary network for the exchange of nutrients, cells, and waste products. This capillary network branches out from arterioles and arteries, in which blood pressure is high, and into venules and veins, in which blood pressure is low (Fig. 4d). A lymphatic network enables drainage of tissue fluid.

Capillaries run parallel to myofibers along the length of the muscle (Fig. 4a). Most are continuous but fenestrated capillaries have been reported (Fig. 3c)^114,115^. Capillaries consist of an inner layer of capillary endothelial cells and an outer layer of contractile smooth muscle (Fig. 4d). Like all endothelial cells, capillary endothelial cells are large cells that connect to each other via tight junctions to form a tubal structure (Figs. 3c and 4d). Molecularly, capillary endothelial cells are defined by carbonic anhydrase (CA4), an enzyme that breaks down CO_2_ into bicarbonate to buffer pH, TCF15, and the pan-endothelial cell marker PECAM1 (CD31) (Table 1)^116,117^. The marker for fenestrated capillary endothelial cells is PLVAP^118^. Capillary proximity to myofibers correlates with local mitochondrial density in those myofibers^119^. Capillary endothelial cells can interact with MuSCs and help maintain MuSC quiescence by expressing the NOTCH ligand DLL4^120^. They survive acute injuries and reassemble into capillaries prior to myogenesis^121^.

Endothelial cells in arteries and arterioles appear narrow and elongated. Arterial endothelial cell markers include GJA5, VWF, and RSG5, whereas arteriolar endothelial cell markers include PSP4, DES, and CNN1 (Table 1)^122–124^. Artery and arteriole endothelial cells maintain vascular resistance. Endothelial cells in venules and veins are relatively short and wide, reflecting the lower blood pressures in veins compared to arteries. Vein endothelial cells express ephrin receptor (EPHB4) protein, neuropilin2 (NRP2) protein, and FABP4 RNA (Table 1)^125–128^. They are essential for hemostasis and blood flow. All vascular endothelial cells derive from a common progenitor and have hardwired cell type-specific gene expression patterns^129^. Accordingly, we list them as different cell types.

Endothelial cells in lymph vessels express PROX1, PDPN, and LYVE1 (Table 1)^130–133^. The number of lymph vessels transiently increases after contraction injuries^131^, possibly to help resolve edema. The mechanisms underlying lymph vessel dynamics in skeletal muscle remain understudied.

Endothelial cells are terminally differentiated. Nevertheless, angiogenesis can occur^134^, potentially driven by endothelial stem cells. Transplantable PECAM1^+^/KIT^+^ endothelial stem cells exist in the lung^135^. Similarly, CD34^+^/LYVE1^low^/PROX1^low^ lymph endothelial stem cells, or lymphangioblasts, exist in fetal human liver^136^. Rare PECAM1^+^/KIT^+^ and CD34^+^/LYVE1^low^/PROX1^low^ cells can be found in muscle scRNA-seq data. Whether these signatures mark endothelial stem cells and lymphangioblasts, respectively, requires further study. Furthermore, a myogenic population in human muscle that is positive for the endothelial cell marker CLEC14A hints at additional unresolved layers of developmental plasticity in the endothelial compartment^137^.

Blood vessels are supported by contractile smooth muscle cells (Figs. 3a and 4d). The most prevalent smooth muscle cell in skeletal muscle is the pericyte, which surrounds the capillaries^138,139^. Pre-capillary pericytes exhibit circular protrusions that wrap around the vessel, mid-capillary pericytes are elongated and spindle-shaped with short branching protrusions, while post-capillary pericytes display a stellate morphology^140^. All express CSPG4 (NG2), ACTA2, and PDGFRB (Table 1)^138,141^. Pericytes maintain vascular integrity and postnatal depletion of CSPG4^+^ cells causes myofiber atrophy^142^. Additionally, pericytes possess ex vivo differentiation potential towards myogenic, adipogenic, fibrogenic, chondrogenic, and osteogenic fates^138^. Pericyte-like cells with remarkably high myogenic potential are called mesoangioblasts^143,144^. Mesoangioblasts express CD146, CD44, and CSPG4 (Table 1)^143,144^. Due to their myogenic capacity and ability to survive in circulation, they are a promising candidate for cell therapy strategies for degenerative muscle diseases^143^.

Arteries and arterioles are lined with vascular smooth muscle myoblasts (VSMs, Fig. 3a). VSMs express MYH11 and ACTA2, but not CSPG4 (Table 1)^139^. VSMs provide a stronger contractile force than pericytes and play crucial roles in maintaining vascular tone and blood pressure^145^. While MYH11-Cre and ACTA2-Cre drivers exist, they have yet to be applied to study skeletal muscle. Lymphatic vessels in skeletal muscle lack a smooth muscle lining and receive hydraulic pressure from myofiber contraction^146^.

#### Immunological microenvironment

Skeletal muscles host immune cells that monitor tissue integrity, including lymphocytes that flag and kill unwanted cells and myelocytes that clear debris and dead cells (Fig. 4e).

The most abundant lymphocyte in the muscle interstitium is the T cell^147^. T cells express CD3E and can be subclassified based on gene expression and function^148^. Most T cells express the ɑβ T cell receptor and either CD4 or CD8 (Fig. 3e). T helper 1 (Th1) cells express CD4 and interferon ɣ (IFNG) and support a pro-inflammatory environment. Prolonged Th1 activity can lead to muscle damage^149^. Th2 cells express CD4 and interleukin-4 (IL4) and support an anti-inflammatory environment^149^. Regulatory T cells (Tregs) express CD4 and FOXP3, and suppress Th1 and Th2 activity (Table 1)^150,151^. Tregs accumulate upon injury and release amphiregulin (AREG) to promote MuSC differentiation^150^. Depletion of FOXP3^+^ cells results in T cell accumulation after acute muscle injury^152^. CD8 T cells exhibit cytotoxicity against target cells^153^. Cytotoxic CD8 T cells express granzymes (GZMB). Myofibers support their function by secreting IL15, which protects CD8 T cells from exhaustion^154^. Consistently, muscle atrophy leads to lower CD8 T cell activity^154^. CD8 T cells accumulate with age and CD8A-deficient mice show impaired muscle regeneration^155,156^. Other cytotoxic lymphocytes are Natural Killer T (NKT) cells and natural killer (NK) cells. NKT cells express T cell markers such as CD3E in addition to NK cell markers such as NK cell granule proteins (NKG7), KLRB1 (NK1.1), and GZMA^157^. Human and mouse NK cells also express NCAM1 and CD16 (FCGR3A), or CD27 and ITGAM, respectively (Table 1)^157,158^. NKT and NK cells accumulate with age and NK cells are recruited upon acute injury in response to IL6^159–161^. Depletion of NK cells results in a loss of IFNG production but only a modest delay in regeneration^162^. Finally, γδ T cells express the γδ T cell receptors. They secrete IL17A in support of muscle regeneration and their depletion results in impaired angiogenesis following ischemic injury^163,164^. In homeostasis, γδT cells are present at low numbers and they accumulate with age^155^.

Unwanted cells can be flagged with antibodies from B cells; small cells with a large nucleus and a cytoplasmic rim sparse in organelles (Fig. 3e)^165^. B cells express CD19, MS4A1 (CD20), and CD22^166^. In response to antigen-presenting cells, they differentiate into plasma cells with an eccentric nucleus and a large cytoplasm (Fig. 3e)^167^. Plasma cells express CD27, CD38, and JCHAIN, but have lost CD19 and CD20 (Table 1)^157,168^. Plasma cells secrete antibodies. B cells are activated by antigen-presenting dendritic cells. Dendritic cells express CD11C (ITGAX), CD209A, and FLT3 (Table 1)^155,169^. They exist in uninjured muscle and are key for the muscle vaccine response^170,171^.

The most prevalent myeloid cell in muscle is the macrophage. Macrophages are large, irregularly shaped phagocytic cells that can be in direct contact with myofibers (Figs. 3e and 4a)^15,172^. They express F4/80 (ADGRE1) and CD11B (ITGAM) and can be subclassified based on genes that were identified following in vitro activation^173,174^. M1 macrophages express CD68, TIM4, and TNFA, whereas M2 macrophages express CD206, CD163, and MRC1 (Table 1)^173,174^. Macrophage depletion impairs regeneration and clearance of apoptotic cells^174^. Moreover, blocking macrophage exocytosis results in reduced membrane repair in myofibers^15^. Macrophages derive from monocytes that transmigrate into the muscle interstitium. Monocytes express CD68 and CSF1R and can be subclassified into classical monocytes expressing CD14 in humans or LY6C in mouse, and non-classical monocytes expressing CD16 in humans or CD43 in mouse (Table 1)^157^. Monocyte depletion causes a reduction in muscle-resident macrophages^175^.

The most abundant immune cell in the human body is the neutrophilic granulocyte, or neutrophil^176^. Neutrophils have a segmented nucleus and, like all granulocytes, contain dense secretory granules filled with digestive enzymes (Fig. 3e)^176^. In the case of neutrophils, those enzymes include lysozyme (LYZ), myeloperoxidase (MPO), and elastase (ELANE)^177,178^. Human and mouse neutrophils express FUT4 and CEACAM8, or LY6G, respectively (Table 1)^155,179,180^. Neutrophils accumulate rapidly upon acute muscle injury, in response to TNFα released from mast cells^179,181^. They aid in clearance of cellular debris by releasing proteases and oxidants^179^. Eosinophilic granulocytes, or eosinophils, express galectin-10 (CLC) and major basic protein (PRG2)^182,183^. Human and mouse eosinophils express lectins SIGLEC8 or SIGLECF, respectively (Table 1)^155,157,183^. Eosinophils drive FAP activation and promote muscle regeneration, whereas excessive eosinophil accumulation impairs regeneration^184,185^. Eosinophils are high in sarcopenia^185^. Basophilic granulocytes, or basophils, are circulatory granulocytes marked by pyrophosphatase ENPP3 and the IgE receptor MS4A2 (Table 1)^157,186^. Mast cells localize to the perimysium and endomysium, and express histamine synthesis genes (HDC) and mast cell proteases (MCPT4-6, TPSB2)^187,188^. Treatment of mice with histamine receptor blockers reduces glycogen synthesis following exercise^188^. Since mast cells are the major source of histamine in skeletal muscle^188^, this indicates an important role for mast cells in muscle homeostasis.

Erythrocytes and thrombocytes are anuclear cells in blood vessel lumens. Erythrocytes express hemoglobins (HBB, HBA1), whereas thrombocytes express pro-platelet basic protein (PPBP) (Table 1)^189,190^. These cell types are common in scRNAseq unless the tissue was perfused, and the marker genes can appear as ambient RNA^191^.

#### Epithelial microenvironment

Skeletal muscle is generally not exposed to the external world and does not need protective epithelium. One exception is the tongue, which is covered with stratified squamous epithelium that is separated from tongue myofibers by fascia^192^. In addition, muscles lining the intraperitoneal and thoracic cavities are covered with mesoderm-derived epithelium called mesothelium, separated from myofibers by lymph vessels^193^. Mesothelium consists of cuboidal mesothelial cells that express mesothelin (MSLN) and the transcription factor WT1, and that exchange biomolecules with the intraperitoneal fluid (Fig. 3d)^194,195^. Germline loss of WT1 leads to diaphragm hernias, suggesting that mesothelium is important for diaphragm structure^195^.

### Cell type abundance

**Fig. 5:**
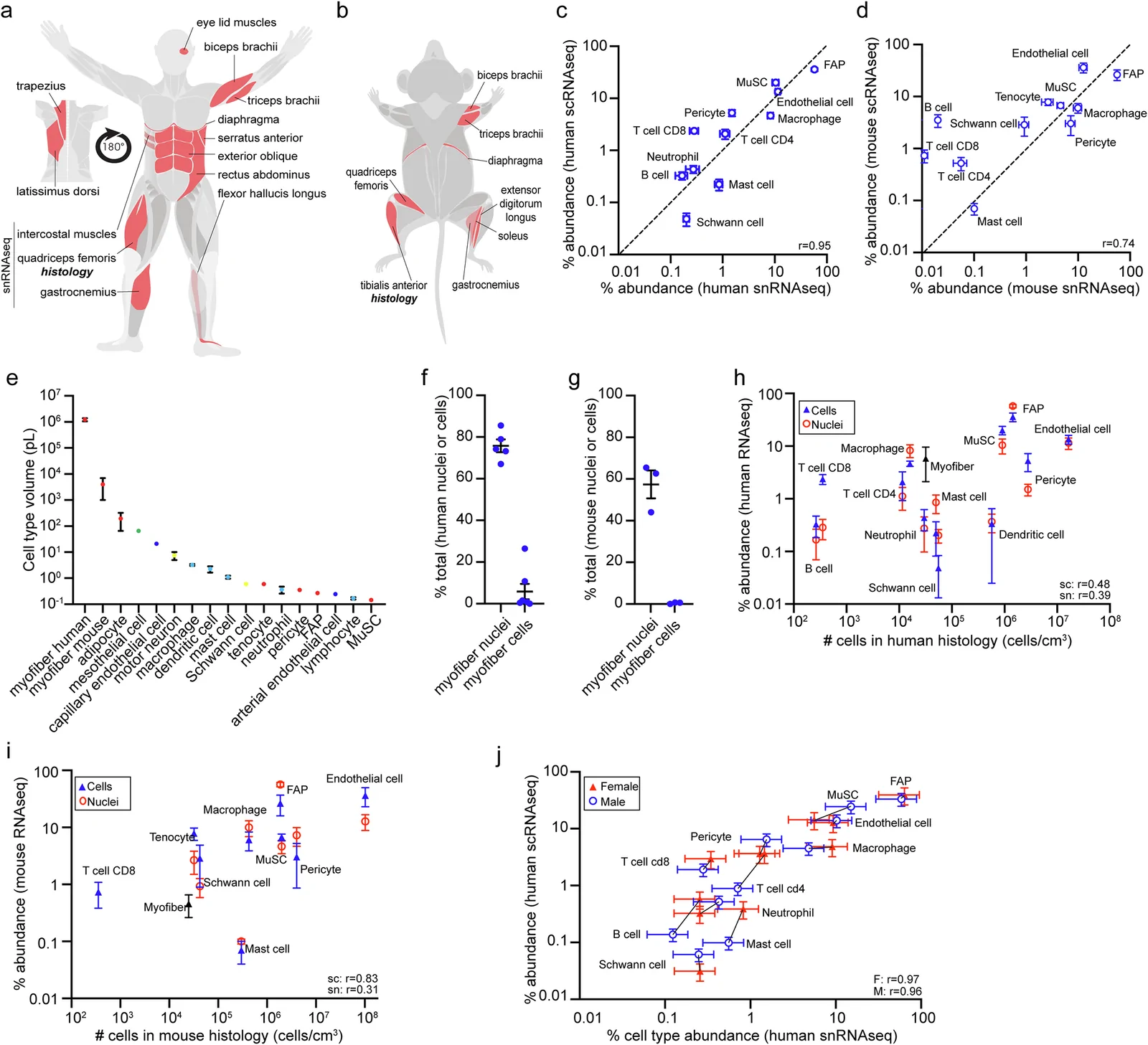
Cell type quantifications. **a**, **b** Schematic of human (**a**) and mouse (**b**) skeletal muscles used in the studies on which subsequent cell type abundance estimates are based. **c**, **d** Relative abundance of indicated cell types in human (**c**) or mouse (**d**) skeletal muscle scRNAseq and snRNAseq analyzes. *n* = 5–7. **e** Graph of cell type volumes. Colors indicate compartments (stromal = red, neuronal = yellow, green = epithelial, blue = endothelial, violet = immune). **f**, **g** Graph of the number of myofiber nuclei and myofiber cells as a percentage of total nuclei or total cells, respectively, for human (**f**) and mouse (**g**) muscle. *n* = 3–7. **h**, **i** For indicated cell types the relative abundances inferred from RNA sequencing data was plotted against absolute cell density in histological sections for human (**h**) and mouse (**i**) muscle. *n* = 3–7. **j** Relative abundance of indicated cell types in human skeletal muscle scRNAseq and snRNAseq analyses, from male and female donors. Datapoints representing the abundance of a cell type in male and female muscles are connected by a line. *n* = 3–4.

We were interested in the abundance of different cell types under homeostasis. For 13 cell types we could retrieve molecular profiles and quantitative histology data, albeit from a diversity of skeletal muscles (Fig. 5a, b). Although this diversity is a source for variation, we decided to include all data to get a rough estimate of cell type abundance across methods. We first compared the relative abundance of these cell types in scRNAseq and snRNAseq, and observed high correlation between the two technologies in human and mouse muscle (Fig. 5c, d, and Supplementary Data). Tissue dissociation (i.e., scRNAseq) results in modest enrichment of B and T cells, which are capable of surviving shear stress. On the other hand, tissue dissociation results in a loss of macrophages and Schwann cells, which are large and may not survive tissue dissociation. Mouse muscle dissociation results in a high yield of Schwann cells, possibly due to the inclusion of peripheral nerves (Fig. 5d).

To compare transcriptomic and histological data, we calculated cell type quantities per cm^3^ of muscle, using cell type volume data (see Supplementary Information, Supplementary Data)^147^. Cell type volumes show a range of 6 orders of magnitude, spanning the 0.5 pL MuSCs to the 1 µL human myofibers (Fig. 5e). This range is largely due to the syncytial nature of the myofibers, which contain hundreds of nuclei. Myofiber nuclei make up more than 70% of all nuclei, yet myofibers make up less than 5% of all cells in muscle tissue (Fig. 5f, g). Cell type abundances in histology positively correlated with those in scRNA-seq or snRNAseq (Fig. 5h, i). Some cell types appear reliably in histology but are absent from transcriptomic data. This includes neurons, which have their soma outside the muscle, eosinophils, Schwann cells, and dendritic cells. In contrast, lymphocytes appear infrequently in histology yet are abundant in transcriptomic data.

Generally, there is high consistency between cell type abundances in human and mouse muscles. Notable exceptions are adipocytes and mast cells, which are more prevalent in human or mouse muscle, respectively. Unlike human histology and snRNAseq, human scRNAseq did not contain adipocytes. This may reflect sensitivity to tissue dissociation. However, the presence of adipocytes in muscle scRNAseq from other mammals suggests that technical artifacts alone cannot fully explain the lack of adipocytes in human muscle scRNAseq^65,196^.

We next parsed the data for variations between male and female samples. Unfortunately, the human histology studies either did not mention the sex of the analyzed samples or used male samples. We were able to plot the relative abundance of cell types in scRNA-seq and snRNA-seq for male and female human muscles (Fig. 5j). Although the small sample sizes preclude meaningful analyzes, there is a trend towards a smaller contribution of immune cells in male muscles compared to female muscles, consistent with a previous study^197^.

Finally, we estimated the total number of each cell type in a human body by extrapolating the abundance quantifications (see Supplementary Information). A reference human has approximately 25 billion MuSCs and 0.83 billion myofibers, in agreement with prior estimates of 15 billion MuSCs and 0.23 billion myofibers^198^. Skeletal muscles also contain 2.5 billion immune cells (2.53E + 09), which is lower than a prior estimate (4E + 10) that included data from non-human primates and extraocular muscles^147^, and 0.45 trillion endothelial cells.

## Conclusions and future directions

We discussed 62 distinct cell types in skeletal muscle in terms of anatomy, molecular signatures, and function. This remarkable diversity largely exists in the interstitium of the muscles, in between myofibers. Whereas most cell types are molecularly distinct entities, in many cases, cell types appear in a continuum, possibly due to shared cell states. The current resource can be a starting point to establish clear cell type definitions and nomenclatures, as has been done for certain immune cell types and neurons^148,199,200^.

Overall, there is a high correlation between relative cell type abundances in immunohistology, scRNAseq, and snRNAseq. However, the different experimental approaches do affect the chance of detection of low-abundant cell types. The study of low-abundant cell types may benefit from optimized tissue dissociation protocols that enable purification, and thus enrichment, of specific cell types. This is common for tissues like the brain, where specific protocols enable isolation of glial cells or neurons^201^, but underexplored for skeletal muscle. Such protocols would go hand in hand with the identification of new cell surface markers to allow for fluorescence-based activated cell sorting of cell populations enriched for rare cell types.

To improve the chance of identification of low-abundant cell types, new cell type markers are needed, as well as algorithms that can identify small cell populations after dimensionality reduction analysis^202^. Improvements in throughput and identification may reveal new low-abundant cell types. Cell type annotation is laborious and relies on expert knowledge. While large language models (LLMs) will likely be able to automate cell type annotation, tests with existing LLMs reveal 80–90% agreement with expert opinion; an excellent first pass but shy of expectations^203^. Our resource will facilitate manual cell type annotation and continued development of automated annotation pipelines.

While the studies discussed in this review offer a glimpse of the incredible cellular diversity present in skeletal muscle, the field needs better tools to investigate the lower-abundance cell types. This includes new Cre drivers for cell lineage tracing and depletion studies to dissect their functions, and new advanced imaging approaches to visualize cell and niche dynamics in vivo^204^. The field also needs a better grasp of cellular heterogeneity across muscles, age, and sex. Moreover, muscles are not homogenous. Since human data are based on small tissue biopsies, different microenvironments may be present in those biopsies depending on how they were obtained. This requires better reporting.

Already, molecular cell atlases are approaching clinical application. The use of cell type benchmarks will facilitate molecular cell autopsies, in which pathological or metastatic cell types can be identified in single-cell transcriptomes from tissue biopsies and traced back to a tissue of origin, as we demonstrated for mouse lemurs^157,205^. This could lead to improved diagnosis and treatments. Resolving the cell types that exist in a given tissue at a given time will enable a deeper understanding of tissue and organ function in health and disease.

### Reporting summary

Further information on research design is available in the Nature Portfolio Reporting Summary linked to this article.

## Supplementary information

Transparent Peer Review file

Supplementary information

Description of Additional Supplementary Files

Supplementary data

reporting summary

## Data Availability

All data are available in the manuscript and in the cited studies.
