# Supplementary material for: A who’s who of cell types in skeletal muscle
Source: Commun Biol. 2026 Sep 3;9:1170. doi: 10.1038/s42003-026-10803-x (PMC13542191; doi:10.1038/s42003-026-10803-x)
Supplement: Supplementary file 1 [file 42003_2026_10803_MOESM1_ESM.pdf]

nature portfolio

Peer Review File

A who’s who of cell types in skeletal muscle

Corresponding Author: Dr Antoine de Morree

This file contains all reviewer reports in order by version, followed by all author rebuttals in order by version.

Version 1:

Reviewer comments:

Reviewer #1

(Remarks to the Author)
The review topic has broad relevance, and I consider it will be of interest not only in the skeletal muscle field but of general interest regarding methodology to define « cell type » numbers whithin specific tissues. The manuscript is clearly written, well-structured, and easy to follow ; I enjoyed reading it.

I think the authors should add a subchapter/or emphasize/distinguish the concepts of cell lineage identity definition "versus" that of cell fate/state (within a certain lineage) definition. I think that the term "cell type" used by the authors refers to "cell fate/state" within a lineage/identity. This was correctly highlighted by the authos in the conclusion as "some cell types appear as a continuum" and dont necessarily change enormously from one another. This is because those « cell types » are actually states/fates of one only cellular identity. I propose to the authors to explain their terminology in the light of these terms.

Reviewer #2

(Remarks to the Author)
In the current article Das Barman summarize data from single cell sequencing and single nuclear sequencing and aim at putting them into perspective regarding skeletal muscle. The authors put a lot of effort into compiling the data from the different sequencing approaches. However, the article reads like a sequence of facts but misses a clear connection of the parts and an introduction into skeletal muscle. Please find here my detailed comments:
1. The article is missing an introduction to skeletal muscle. It begins with an introduction to the sequencing techniques and their advantages and disadvantages. This is an important topic for the article but should not be in the first paragraph.
2. From the abstract it is not clear if it is a review article or an original research article. Sentences like “we define 63 known cell types” suggest that it is a research article. Please rephrase the abstract corresponding to the changes in the manuscript and make sure it is clear to the reader that this is really a review article.
3. The article is missing a clear description how the different cell types in skeletal muscle are interacting – this is important to have this in a separate chapter and not as a half sentence in the chapter describing the different cell types. This is especially important since the title is cellular microenvironments…
4. The different cell types identified by the authors are described in detail regarding their marker expression and sometimes also their function. The amount of facts provided by the authors are hard to grasp for the reader in that form. I would recommend to generate a table with the main facts about the cell types and the markers used. That would enhance the quality and usability of the provided information a lot.
5. It is not clear to me if the graphs provided in Fig. 3 are for muscle under resting conditions or after injury and which muscle groups were used to generate the graphs. Please add more information here. Also a more detailed description on how you generated the graphs is required – although it is not an original article. This could be done as supplemental information or in a paragraph together with the part describing the advantages/disadvantages of the sequencing methods. It is also not clearly visible in the figure when human and when human and murine data are plotted.
6. Figure 2 does not provide any additional information – at least for me. Please remove it from the article or embed it better into the writing.
7. Is Figure 1 generated by the authors or a modification from a published illustration. If the latter is the case the citation is missing. Please include.
8. The authors write about the differences between mice and humans in general and how important it is to pay attention to it. However, in the description of the different cell types this aspect is falling short. Please include the respective information

here.
9. The authors focus on human and mouse data. However, they regularly cite their own work on lemurs out of the context. This is inappropriate. Please remove these self-citations which are out of context there. Alternatively include a paragraph on other animal models and discuss those (and not only the data generated by the de Morree group).
10. Figure 4 and 5 have no connection to the text. Please either remove them or embed them better into the writing.
11. The authors put data from different muscle groups, ages, sex into one analysis (Fig. 3). In my opinion this is not appropriate. Please correct at least for muscle type and age.

Reviewer #3

(Remarks to the Author)
I co-reviewed this manuscript with one of the reviewers who provided the listed reports. This is part of the Communications Biology initiative to facilitate training in peer review and to provide appropriate recognition for Early Career Researchers who co-review manuscripts.

Version 2:

Reviewer comments:

Reviewer #1

(Remarks to the Author)
The authors clearly responded to my points and modified the manuscript that I think can be published.

Reviewer #2

(Remarks to the Author)
The authors addressed all concerns I raised in the first round of review. The manuscript nicely improved and will be an asset to the community. The inclusion of additional chapters and rewriting some paragraphs improved the reading flow and enhanced the understandability. Furthermore, including a table for the different cell types and the respective marker expression will make this review very useful. I congratulate the authors to a nice review on the different cell types in skeletal muscle.

Reviewer #3

(Remarks to the Author)
I co-reviewed this manuscript with one of the reviewers who provided the listed reports. This is part of the Communications Biology initiative to facilitate training in peer review and to provide appropriate recognition for Early Career Researchers who co-review manuscripts.

Open Access This Peer Review File is licensed under a Creative Commons Attribution 4.0 International License, which permits use, sharing, adaptation, distribution and reproduction in any medium or format, as long as you give appropriate credit to the original author(s) and the source, provide a link to the Creative Commons license, and indicate if changes were made.
In cases where reviewers are anonymous, credit should be given to 'Anonymous Referee' and the source.
The images or other third party material in this Peer Review File are included in the article's Creative Commons license, unless indicated otherwise in a credit line to the material. If material is not included in the article's Creative Commons license and your intended use is not permitted by statutory regulation or exceeds the permitted use, you will need to obtain permission directly from the copyright holder.
To view a copy of this license, visit https://creativecommons.org/licenses/by/4.0/

RESPONSE TO REFEREES' COMMENTS

GENERAL RESPONSES

We thank the Referees for their careful reading of the manuscript and for their constructive comments to improve the manuscript overall. Below we have responded in a point-by-point fashion to each comment by each Referee. We have made several major revisions to the manuscript in response to Referee suggestions:

- We wrote new chapters on the topics of cell types and states, and of cell micro-environments and interactions.
- We created a new Figure 2 to illustrate the differences between cell types and cell states, and a new Figure 4a to illustrate molecular signatures across myonuclei that reflect interactions between the myofibers and other cell types.
- We made textual changes to the manuscript to focus more on the discussion of cell types and less on the discussion of abundance. This includes moving the chapter on the topic of cell abundance to the back of the manuscript and including an extended Supplementary Notes section on how we compared cell abundance data.

POINT-BY-POINT RESPONSES TO REFEREES' COMMENTS

Editorial comments

"please make it more clear that this is indeed a Review and that the cell types classified here were characterized based on a literature meta-analysis."

We have adjusted the wording in the abstract and the introduction to emphasize that all cell types have been reported in the literature.

"please ensure you convert the discussion into more of a scholarly discussion of skeletal muscle cell types, which is complemented by your own analysis, with a detailed methodology in a note in the suppl material."

We have moved the chapter with the topic of cell type abundance to the back of the manuscript (now page 12, line 456). We also added text to the Supplemental Materials on our approach (page 3, line 25). In brief, we compared published studies that used different measures of quantification (i.e. cells per area, per volume, per field, per reference cell type). To enable comparisons across these different measures, we applied a standardized calculation method to calculate cells per volume of muscle.

REFEREE #1

"The review topic has broad relevance, and I consider it will be of interest not only in the skeletal muscle field but of general interest regarding methodology to define « cell type » numbers within specific tissues. The manuscript is clearly written, well-structured, and easy to follow; I enjoyed reading it."

We thank the Referee for the positive words.

"I think the authors should add a subchapter/or emphasize/distinguish the concepts of cell lineage identity definition "versus" that of cell fate/state (within a certain lineage) definition. I think that the term "cell type" used by the authors refers to "cell fate/state" within a lineage/identity. This was correctly highlighted by the authors in the conclusion as "some cell types appear as a continuum" and dont necessarily change enormously from one another. This is because those « cell types » are actually states/fates of one only cellular identity. I propose to the authors to explain their terminology in the light of these terms."

We have added a new chapter immediately after the introduction to discuss cell types, states, and lineages. This chapter is supported by a new figure to illustrate the differences between cell types and cell states (Figure 2). Moreover, this new chapter allowed us to better integrate Figure 3 (previously Figure 2) into the text by discussing cell lineage. In addition, the new chapter led us to re-examine our list of cell types and we decided to remove one cell type, the reticular cell, since the discriminating factor is only a single molecular feature.

The new chapter is on page 3, line 89-119. The new Figure 2 is copied below:

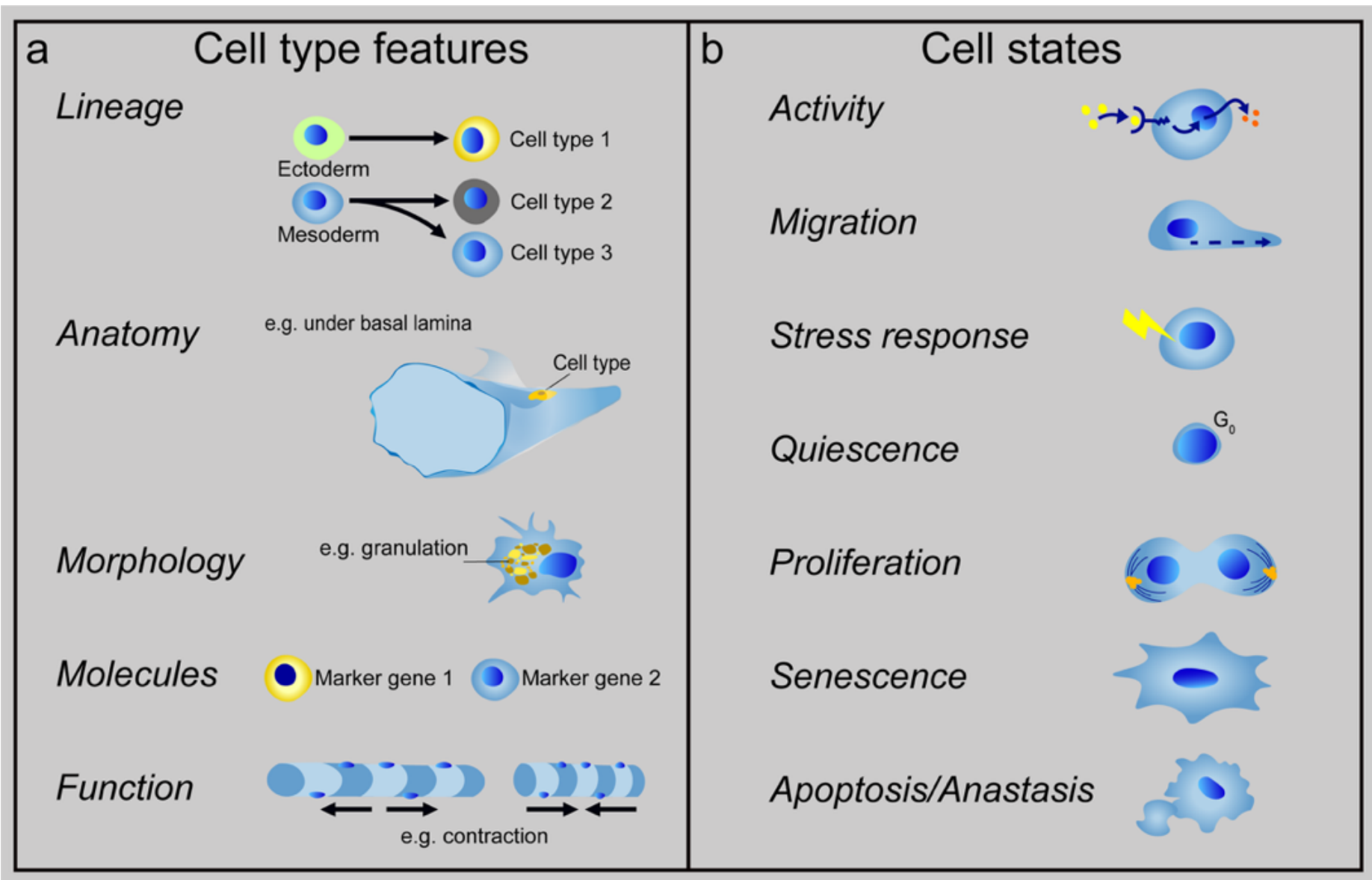

Figure legend: a) Cells possess identifiable features that reflect their identity, or type. These features are hardwired and stable. b) Cells can engage in various processes that reflect their current activity, or state. Cell states are mostly transient and reversible, though they can be irreversible.

<u>REFEREE #2</u>

"In the current article Das Barman summarize data from single cell sequencing and single nuclear sequencing and aim at putting them into perspective regarding skeletal muscle. The authors put a lot of effort into compiling the data from the different sequencing approaches."

We thank the Referee for recognizing the effort we put into compiling all data.

"1. The article is missing an introduction to skeletal muscle. It begins with an introduction to the sequencing techniques and their advantages and disadvantages. This is an important topic for the article but should not be in the first paragraph."

We have rewritten the first and last paragraph of the Introduction to provide more introduction to skeletal muscle tissue.

The rewritten paragraphs are at the top of page 2, line 27-36, and of page 3, line 71-87.

"2. From the abstract it is not clear if it is a review article or an original research article. Sentences like "we define 63 known cell types" suggest that it is a research article. Please rephrase the abstract corresponding to the changes in the manuscript and make sure it is clear to the reader that this is really a review article."

We have made adjustments to the wording in the abstract to better highlight that our research is entirely based on published literature and that we provide cell type definitions based on cell features reported in the literature.

The sentence in the abstract now reads: "Using skeletal muscle as a model, we discuss basic principles for cell type classification, summarize 62 unified definitions of cell types in terms of lineage, molecular signatures, and function from the literature, and discuss how these cell types contribute to muscle function." Page 1, line 15.

"3. The article is missing a clear description how the different cell types in skeletal muscle are interacting – this is important to have this in a separate chapter and not as a half sentence in the chapter describing the different cell types. This is especially important since the title is cellular microenvironments… "

We have included a new chapter on cell microenvironments in which we highlight the importance of cell type interactions. This also allowed us to better embed Figure 4 into the text. Needless to say, discussing how all cell types interact is a Review topic on its own and goes beyond the scope of our manuscript. Instead, we discuss cell type interactions from the perspective of the myofiber.

The new chapter is on page 4, line 120-134. The new Figure panel is below:

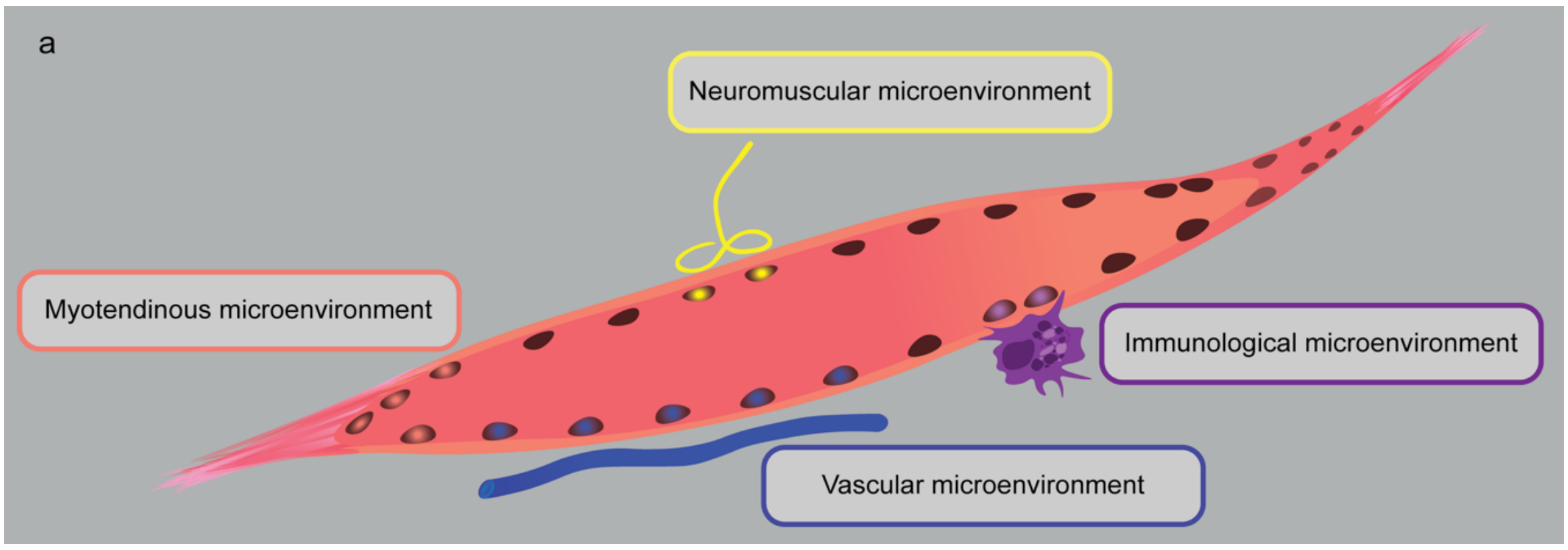

Figure legend: Schematic overview of a myofiber and its peripheral myonuclei, with four key anatomical microenvironments highlighted: the myotendinous, neuromuscular, vascular, and immunological microenvironments. Myonuclei near the site of interaction with these microenvironments show distinct transcriptional signatures, indicated in the figure with distinct coloration.

"4. The different cell types identified by the authors are described in detail regarding their marker expression and sometimes also their function. The amount of facts provided by the authors are hard to grasp for the reader in that form. I would recommend to generate a table with the main facts about the cell types and the markers used. That would enhance the quality and usability of the provided information a lot."

We have embedded Table 1 into the main manuscript and included information on cell function, anatomy, and markers.

Table 1 is on pages 20 and 21.

"5. It is not clear to me if the graphs provided in Fig. 3 are for muscle under resting conditions or after injury and which muscle groups were used to generate the graphs. Please add more information here. Also a more detailed description on how you generated the graphs is required – although it is not an original article. This could be done as supplemental information or in a paragraph together with the part describing the advantages/disadvantages of the sequencing methods. It is also not clearly visible in the figure when human and when human and murine data are plotted."

We thank the Referee for pointing this out. We have now specified in the manuscript that we only included data obtained from homeostatic conditions in our cell type abundance comparisons. Page 12, line 457.

We expanded the Supplementary Notes with details on how we used the free webtool CellxGene to analyze published transcriptomic data and how we applied a standardized calculation to all published quantitative histology data. We used Graphpad Prism to render the graphs. Page 3, line 48-59.

In Figure 5 (formerly Figure 3), we have now included species information in all graph axis labels. Moreover, we now graphically highlight the different muscles on which the data analyses are based in Figure 5a,b. Page 19.

"6. Figure 2 does not provide any additional information – at least for me. Please remove it from the article or embed it better into the writing."

We have added a new chapter on cell type definitions, states, and lineages, which enabled us to better embed Figure 3 (previously Figure 2) into the manuscript See also our response to comment 1 of Referee 1.

The new chapter is on page 3 and 4, line 89-119.

"7. Is Figure 1 generated by the authors or a modification from a published illustration. If the latter is the case the citation is missing. Please include."

Figure 1, like all other schematic Figures, is indeed drawn in Adobe Illustrator. Accordingly, no citation is needed. We added a section to the Supplemental Notes on our approach to drawing these figures. Page 3, line 61-66.

"8. The authors write about the differences between mice and humans in general and how important it is to pay attention to it. However, in the description of the different cell types this aspect is falling short. Please include the respective information here."

We would like to point out that was by no means our objective to point out differences between humans and mice and discuss their importance. Instead, we highlight those differences as an important note to researchers who seek to annotate cell types in their transcriptomic data. Accordingly, we list species-specific cell type marker genes in Table 1 and highlight some of them in the text. While species-specific marker genes are known for many cell types, there are few studies that offer direct comparisons of cell types across species in terms of function. We believe an in-depth discussion of such species-specific analyses of cell type function is out of scope for the current manuscript.

We do highlight that some cell types have not (yet) been described in both species. The simplest explanation for this is the limited number of published studies on these particular cell types. We have made a clarification in the text that those cell types that have been reported in only one species thus far are understudied, by adding the words "The remaining cell types are *understudied*". This text is now part of the new chapter on cell types and states, on page 4, line 113-119.

"9. The authors focus on human and mouse data. However, they regularly cite their own work on lemurs out of the context. This is inappropriate. Please remove these self-citations which are out of context there. Alternatively include a paragraph on other animal models and discuss those (and not only the data generated by the de

Morree group).”

We absolutely agree with the Referee that a discussion focused on the biology of human and mouse cells should refrain from mentioning the biology of cells in other species. Accordingly, we would like to stress that we *do not* refer to our mouse lemur studies to discuss mouse lemur *biology*. Rather, we refer to the work to make a *technical* point, namely that the absence of adipocytes in human muscle single cell RNA sequencing experiments cannot fully be explained by a technical artifact of the method, because such cells are in fact present in single cell RNA sequencing experiments from mouse lemur muscle. We think that this is an important, and thus appropriate, point to make in a discussion of cell type abundances across data sets generated with different technologies. We have amended the citation to further stress that we are interested in the *technical*, rather than the *biological*, perspective. In addition, per the Referee's suggestion, we included a reference on pig muscle that supports the same point. The citation now reads: “Unlike human histology and snRNAseq, human scRNAseq did not contain adipocytes. This may reflect sensitivity to tissue dissociation. However, the presence of adipocytes in muscle scRNAseq from other mammals suggests that technical artifacts alone cannot fully explain the lack of adipocytes in human muscle scRNAseq.” Page 12, line 479-484.

Similarly, we cite our mouse lemur atlas in the Future Perspectives section, *not* to make a *biological* point, but to highlight a novel *technical* perspective, namely that molecular cell atlases when combined with cell type references could be developed into a diagnostics tool. This is based on a serendipitous finding in our mouse lemur work where one animal suffered from metastatic endometrial cancer, and the cell atlas could reveal both the metastatic cancer cells and the cell type and tissue of origin. Again, we think that this is an important perspective on the *technical* impact of cell type references, such as our manuscript, and cell atlases. We have shortened the citation to highlight our interest in the technical perspective. Page 14, line 531-536.

“10. Figure 4 and 5 have no connection to the text. Please either remove them or embed them better into the writing.”

We have embedded Figure 4 into a new chapter on cellular microenvironments and cell type interactions. In addition, we modified the first panel of Figure 4 to illustrate molecular signatures of myofiber interactions. See also our response to comment 3. Page 4, line 120-134.

We have integrated Figure 5 with the previous Figure 3 to make better use of the schematic visuals to highlight which muscles are used for which analyses and to better embed the Figure into the text. Page 12, line 456-496.

“11. The authors put data from different muscle groups, ages, sex into one analysis (Fig. 3). In my opinion this is not appropriate. Please correct at least for muscle type and age.”

We absolutely agree with the Referee that analyzing data across different muscles introduces variability and potential complications. This is why we were upfront about integrating data from different muscles. Our objective here was to provide a rough estimate of cell type numbers and we opted for statistical power over biological separation. The reality is that, especially when it comes to human data, the sourced muscles are highly diverse between studies and between donors in a single study; something that can be traced back to the often-opportunistic sourcing of intraoperative biopsies. In addition, human samples come in a large range in age and demographics that skews towards older individuals. While mouse studies generally do not suffer from a large diversity in sourced muscles, these are frequently based on pooled muscles from male mice. The sole exception is the histology analysis, which we were able to restrict to single muscles (quadriceps for human, TA for mouse).

We have now added text to the Supplementary Notes to clarify which muscles were used for the cell abundance comparisons, and we added a tab to Supplementary Table 1 on the muscles used in each of the transcriptomic studies. We also added text to the main manuscript to clarify our rationale for providing the cell type abundance estimates. Moreover, we have amended Figure 5 (formerly Figures 3 and 5) to better highlight which abundance estimates are based on data from which muscles.

Finally, we were able to separate male and female data for human muscles and plotted those in Figure 5j.

REVIEWER #3

"I co-reviewed this manuscript with one of the reviewers who provided the listed reports. This is part of the Communications Biology initiative to facilitate training in peer review and to provide appropriate recognition for Early Career Researchers who co-review manuscripts."

Thank you for participating in this program and taking the time to thoroughly read and critique our manuscript.

POINT-BY-POINT RESPONSES TO REFEREES' COMMENTS

REFEREE #1

"The authors clearly responded to my points and modified the manuscript that I think can be published."

We thank the Referee for recommending our manuscript for publication.

REFEREE #2

"The authors addressed all concerns I raised in the first round of review. The manuscript nicely improved and will be an asset to the community. The inclusion of additional chapters and rewriting some paragraphs improved the reading flow and enhanced the understandability. Furthermore, including a table for the different cell types and the respective marker expression will make this review very useful. I congratulate the authors to a nice review on the different cell types in skeletal muscle."

We thank the Referee for the positive words and recognizing that our manuscript will be of use to the community.

REVIEWER #3

"I co-reviewed this manuscript with one of the reviewers who provided the listed reports. This is part of the Communications Biology initiative to facilitate training in peer review and to provide appropriate recognition for Early Career Researchers who co-review manuscripts."

Thank you again for making time to participate in the review of our manuscript.
