# Supplementary material for: A who’s who of cell types in skeletal muscle
Source: Commun Biol. 2026 Sep 3;9:1170. doi: 10.1038/s42003-026-10803-x (PMC13542191; doi:10.1038/s42003-026-10803-x)
Supplement: Supplementary file 2 [file 42003_2026_10803_MOESM2_ESM.pdf]

Supplementary information to:

A who's who of cell types in skeletal muscle

Shubhangi Das Barman[1,*], Zofija Frimand[1,*], M. Pilar J. Stella[1,*], Anne-Sofie Clausen[1], Sierra L. W. Olsen[1], Antoine de Morree[1,#]

1. Department of Biomedicine, Aarhus University, Aarhus, Denmark

* Equally contributing

# Correspondence to: demorree@biomed.au.dk

Supplementary Data Legends

Supplementary Data: primary data underlying cell type quantifications. Overview of the raw data corresponding to Figure 5, including histological measurements, volumetric analyses, compartmental distribution, overall cell type abundance, relative abundance of distinct cell populations in male and female samples, and FACS validation analyses.

Supplementary Notes

Cell Type Definitions

To name cell types, we used Cell Ontology, a curated database of cell type names[1].

Cell type abundance calculations

Cell type quantifications are common in literature but often presented in different formats (e.g. cells per area, per field, per volume, or per reference cell type) making comparison challenging. We obtained quantitative histology data and applied a standardized calculation to compare cell type abundance data. To this end, we followed the approach taken by Sender et al[2]. We conducted a thorough review for quantitative histology studies that 1) used established cell type marker antibodies and 2) provided quantitative data on the cell type abundance (either per surface area or per reference cell type). We searched using PubMed, Google, Google Scholar, Gemini AI, and Chat GPT using keywords related to measurement (e.g. "population," "number," "density," "histology,") tissue (e.g. "skeletal muscle," "quadriceps") and cell type (e.g. "muscle stem cell," "satellite cell," "pax7"). We focused on data from the quadriceps and tibialis anterior muscle, the predominantly studied human and mouse muscle, respectively. Cell type quantifications were assessed and transformed into cell density estimates based on tissue volume (i.e., number of cells per cm^3 of tissue). Cell density estimates were calculated from histological quantifications of the number of cells (n) per area unit (in cm^2) using the thickness of the sample (in cm) and the diameter of a single cell (in cm), based on the formula: cells/cm^3 = mean cell type # / (area in cm^2 x (section thickness in cm + cell diameter in cm)[2].

$$cell\ density\left(\frac{cells}{cm3}\right) = \frac{n(cells)}{area\ (cm2) * (section\ thicknes\ (cm) + cell\ diameter\ (cm))}$$

We extrapolated cell densities to the reference human (a healthy male, 176 cm tall, 73 kg heavy, and 20-30 years old[3]). Skeletal muscles comprise approximately 40% of body weight[4]. Lean skeletal muscle density is 1.06 kg/L[5]. Therefore, the reference human has 27.5 L of skeletal muscles. We experimentally benchmarked cell volume and cell abundance for MuSCs and FAPs by fluorescence-activated cell sorting and hemocytometer analysis (Supplementary Table 1).

We analyzed and annotated published data sets generated with 10x genomics technology and obtained from different human and mouse muscles under homeostasis (supplementary Table 1)[6–15]. Data were explored with the open access webtool CellxGene

(cellxgene.cziscience.com/)[16] and cell types were assigned based on the marker profiles in Table 1. Relative cell type numbers were calculated as a percentage of total cells in the dataset or as a percentage of total nuclei minus myonuclei. We calculated cell type abundances per study, rather than per donor, because of the large variations in donor muscles, demographics, and cells/nuclei sequenced. Surprisingly, standard deviations across studies are minor suggesting good reproducibility of cell type abundances across studies despite the variability in sourced muscles, donor demographics, and sample quality.

All graphs were rendered in Graphpad Prism 11. Each data point reflects the average of multiple studies.

Figure drawing

Original figures were drawn to highlight anatomical and morphological features while providing an overview of the location of cell types in their respective microenvironments. Illustrations were created using Adobe Illustrator 2024 combined with Wacom One drawing pad (CTC4110WL). All illustrations are original, drafted based on existing literature, and further developed for clarity based on scientific discussions among all authors.

Statistics

Non-linear regression analyses were performed with Graphpad Prism 11.

Supplementary References

1. Diehl, A. D. *et al.* The Cell Ontology 2016: enhanced content, modularization, and ontology interoperability. *J Biomed Semantics* **7**, 44 (2016).
2. Sender, R. *et al.* The total mass, number, and distribution of immune cells in the human body. *Proc Natl Acad Sci U S A* **120**, e2308511120 (2023).
3. ICRP. *ICRP Publication 89: Basic Anatomical and Physiological Data for Use in Radiological Protection: Reference Values*. (SAGE Publications Limited, 2003).
4. Frontera, W. R. & Ochala, J. Skeletal muscle: a brief review of structure and function. *Calcif Tissue Int* **96**, 183–195 (2015).
5. Etchison, W. C. Letter to the editor response. *Sports Health* **3**, 499 (2011).
6. Kedlian, V. R. *et al.* Human skeletal muscle aging atlas. *Nat Aging* **4**, 727–744 (2024).
7. Xu, C. *et al.* Automatic cell-type harmonization and integration across Human Cell Atlas datasets. *Cell* **186**, 5876–5891.e20 (2023).
8. Perez, K. *et al.* Single nuclei profiling identifies cell specific markers of skeletal muscle aging, frailty, and senescence. *Aging (Albany NY)* **14**, 9393–9422 (2022).
9. De Micheli, A. J., Spector, J. A., Elemento, O. & Cosgrove, B. D. A reference single-cell transcriptomic atlas of human skeletal muscle tissue reveals bifurcated muscle stem cell populations. *Skelet Muscle* **10**, 19 (2020).
10. He, S. *et al.* Single-cell transcriptome profiling of an adult human cell atlas of 15 major organs. *Genome Biol* **21**, 294 (2020).
11. Tabula Sapiens Consortium* *et al.* The Tabula Sapiens: A multiple-organ, single-cell transcriptomic atlas of humans. *Science* **376**, eabl4896 (2022).
12. Eraslan, G. *et al.* Single-nucleus cross-tissue molecular reference maps toward understanding disease gene function. *Science* **376**, eabl4290 (2022).

13. Tabula Muris Consortium *et al.* Single-cell transcriptomics of 20 mouse organs creates a Tabula Muris. *Nature* **562**, 367–372 (2018).
14. Tabula Muris Consortium. A single-cell transcriptomic atlas characterizes ageing tissues in the mouse. *Nature* **583**, 590–595 (2020).
15. Ham, A. S. *et al.* Single-nuclei sequencing of skeletal muscle reveals subsynaptic-specific transcripts involved in neuromuscular junction maintenance. *Nat Commun* **16**, 2220 (2025).
16. CZI Cell Science Program *et al.* CZ CELLxGENE Discover: a single-cell data platform for scalable exploration, analysis and modeling of aggregated data. *Nucleic Acids Res* **53**, D886–D900 (2025).
