# Supplementary material for: A who’s who of cell types in skeletal muscle
Source: Commun Biol. 2026 Sep 3;9:1170. doi: 10.1038/s42003-026-10803-x (PMC13542191; doi:10.1038/s42003-026-10803-x)
Supplement: Supplementary file 3 [file 42003_2026_10803_MOESM3_ESM.pdf]

Description of Additional Supplementary Files

File name: Supplementary Data 1

Description: primary data underlying cell type quantifications. Overview of the raw data corresponding to Figure 5, including histological measurements, volumetric analyses, compartmental distribution, overall cell type abundance, relative abundance of distinct cell populations in male and female samples, and FACS validation analyses.
